# Persistent residual ellipticity in waveplate-compensated polarization-resolved SHG microscopy on a commercial multiphoton microscope

**DOI:** 10.1371/journal.pone.0357256

**Published:** 2026-11-13

**Authors:** David Nguyen, Jeffrey P. Wilde, Virginie Uhlmann, Daniel James Smith, Justine Kusch-Wieser, Valeria Zanrè, Jakob Schwiedrzik, Gábor Csúcs

**Affiliations:** 1 Scientific Center for Optical and Electron Microscopy (ScopeM), ETH Zürich, Zürich, Switzerland; 2 Department of Electrical Engineering, Stanford University, Stanford, California, United States of America; 3 Department of Molecular Life Sciences, University of Zürich, Zürich, Switzerland; 4 Laboratory for High Performance Ceramics, Empa Dübendorf, Dübendorf, Switzerland; Commonwealth Scientific and Industrial Research Organisation, AUSTRALIA

## Abstract

Polarization-resolved second harmonic generation microscopy provides structural information about non-centrosymmetric biological samples such as collagen. It involves illuminating the sample with a focused laser beam having a variable linear polarization angle and recording the second harmonic signal as a function of this angle. However, accurate linear polarization control is challenging due to ellipticity introduced by reflections from mirrors and dichroic mirrors in the optical path. Waveplate-based compensation has emerged as the standard approach to address these distortions, but its effectiveness for quantitative measurements remains incompletely characterized. Here, we reproduce an established automated waveplate compensation method, based on a rotating half-waveplate combined with a compensating quarter-waveplate, on a commercial Leica TCS SP8 MP multiphoton microscope, which is the same type of instrument the method was originally developed on. Despite significant effort, we consistently observed substantial unwanted residual polarization ellipticity, with amplitudes up to 0.24, persisting under optimal waveplate configurations. Our simulation analysis suggests that this limitation may arise from wavelength-dependent dichroic mirror birefringence combined with the broad spectral bandwidth (10 nm to 20 nm full width at half maximum) of femtosecond laser pulses. While the approach investigated here can compensate a single wavelength, different spectral components within the pulse experience different phase retardations from wavelength-dependent optical elements, potentially resulting in residual ellipticity that cannot be eliminated. Our simulations qualitatively reproduced key features of the experimental observations. These findings have important implications for quantitative polarization-resolved second harmonic generation microscopy and suggest that alternative approaches, including specimen rotation or picosecond laser sources with narrower bandwidth, should be investigated for applications requiring precise polarization control. To facilitate community investigation of these effects, we provide open-source analysis code and simulation files.

## Introduction

Second harmonic generation (SHG) is a nonlinear optical process in which two identical photons combine to form a single photon at exactly twice the incident frequency [1]. An essential requirement for SHG is that the medium is non-centrosymmetric [2]. This symmetry requirement has made SHG particularly valuable for biological imaging, where non-centrosymmetric structures such as collagen can be studied without labels [3,4]. The polarization dependence of SHG provides structural information about fiber orientation and molecular organization [5]. Polarization-resolved measurements are performed by illuminating the sample with linear polarization and varying the relative angle between the fundamental beam polarization and the specimen orientation while recording SHG intensity [6,7]. However, achieving accurate linear polarization control is challenging due to ellipticity introduced by reflections from mirrors and dichroic mirrors, and polarization distortions from high numerical aperture (NA) objectives [8]. In commercial multiphoton microscope systems, where the optical layout is largely fixed and polarization filtering immediately before the objective is generally not possible, polarization distortions must be actively managed. While the use of lower NA objectives minimizes these distortions, residual ellipticity from optical elements in the beam path must still be addressed. Two approaches exist: compensating with waveplates to enable variable incident polarization [9], or performing measurements at a fixed incident polarization state (chosen to introduce minimal ellipticity) while rotating the specimen to vary the relative orientation [10]. The waveplate compensation approach uses a linearly polarized laser beam passed through a quarter-waveplate (QWP) followed by a half-waveplate (HWP). When a principal axis of the QWP is aligned with the incident polarization, the beam remains linearly polarized and the HWP can be rotated to control the polarization angle on the sample. The QWP can then be adjusted to compensate for ellipticity introduced by optical elements between the waveplates and the specimen, with the goal of maintaining optimal linear polarization across the full range of HWP rotation angles. Specimen rotation, however, is complicated by alignment challenges, as the rotation axis is typically not coaxial with the imaging field, making waveplate compensation the more commonly adopted approach.

Building on the automated waveplate compensation method for commercial microscopes developed by Romijn et al. [11], we implemented their approach on a Leica TCS SP8 MP—the same instrument type tested in their work. Our primary aim was thus to reproduce this established compensation method on the same class of commercial instrument, and to document its practical performance for the community. We improved system characterization by implementing a fast motorized polarization analyzer with a photodiode detector, enabling high-resolution ellipticity maps with double the waveplate angular sampling resolution and significantly reduced acquisition time. Our systematic measurements revealed that compensation primarily symmetrizes the angle-dependent ellipticity, but substantial residual ellipticity persists under optimal configurations. We tentatively attribute this effect to a combination of wavelength-dependent dichroic retardance [12] with the broad spectral bandwidth of femtosecond lasers, which we propose may reflect a limitation of the waveplate compensation approach. In this work, we present our hardware and software implementation, systematic ellipticity measurements at the entrance port and sample plane, and computational simulations to explore potential physical mechanism underlying these observations. We report our results as a cautionary note for groups considering this approach on similar commercial systems.

## Materials and methods

The polarization rotation and compensation module was implemented following Romijn et al. [11] and consists of a QWP followed by a HWP in the fundamental beam path before the microscope (Fig 1). The fundamental wavelength was 880 nm, generated by a Ti:Sapphire femtosecond laser (Mai Tai, Spectra-Physics). This waveplate compensation approach was originally developed by Chou et al. [9] using a HWP-QWP configuration. The waveplate ordering is critical as it determines the mathematical formulation of the compensation curves. The QWP-HWP order offers the practical advantage that the QWP can be fixed while HWP rotation controls the polarization angle. Both waveplates were mounted in motorized rotation stages (Table 1). Implemented in this way, the compensation module requires approximately 70 mm of optical path length, plus additional space if enclosed for laser safety. We positioned it after beam conditioning optics and immediately before the microscope entrance port (see S1 Fig). Due to variability in beam delivery between commercial systems, we do not provide detailed mounting specifications. The waveplates were positioned approximately perpendicular to the beam axis and centered in the beam path. As in the calibration procedures of Romijn et al. [11] and Chou et al. [9], all polarization measurements reported in this work were performed at the sample plane with the objective lens removed, and no objective lens was therefore present in the beam path for any measurement.

**Fig 1:**
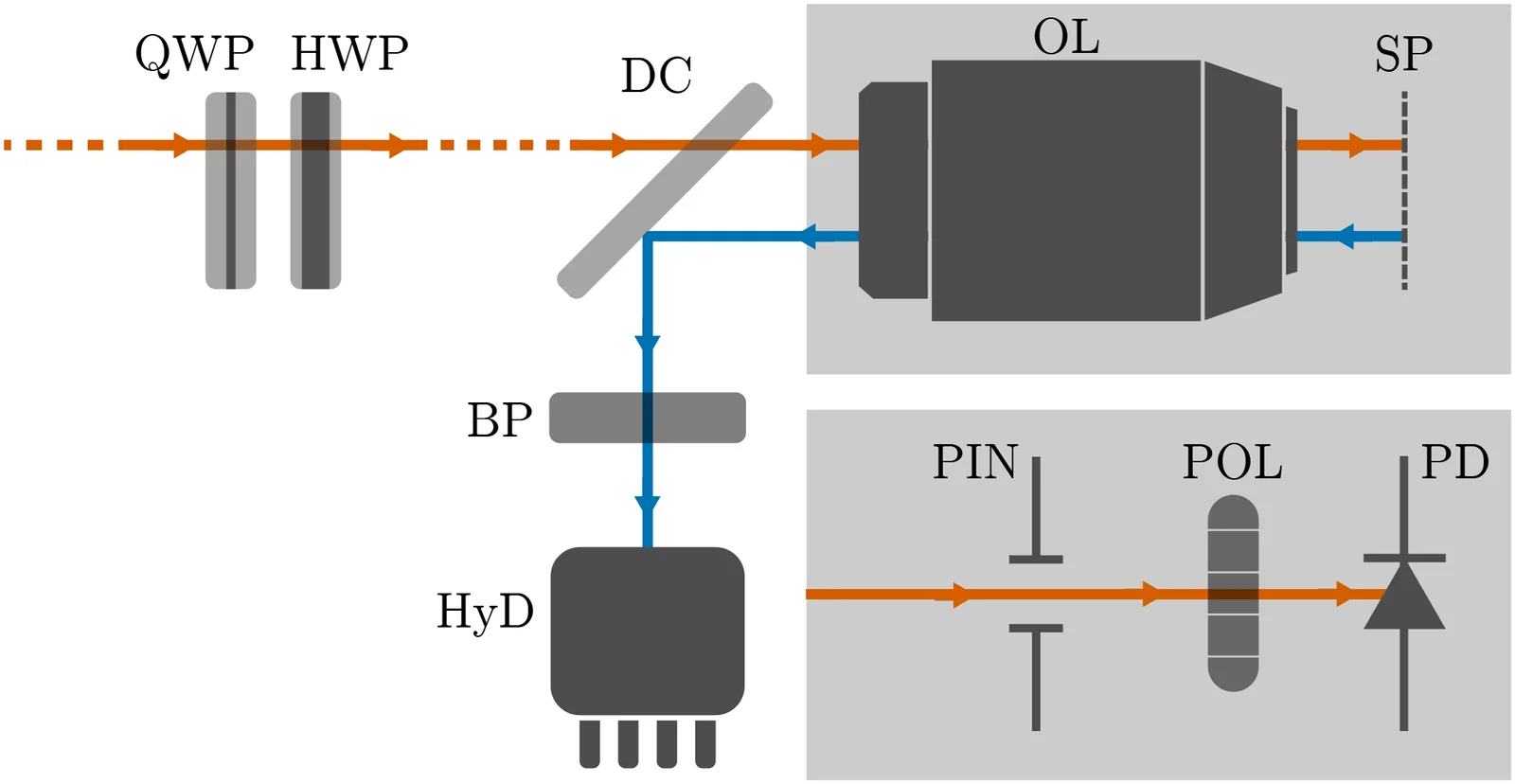
Optical layout. Fundamental beam from the laser tuned at 880 nm (orange) passes through the beam conditioning optics (not shown, indicated by dashed line) before entering the compensation module made of a quarter-waveplate (QWP) and half-waveplate (HWP). The beam then passes through the microscope optics (dashed section) to the objective lens (OL) and is focused at the sample plane (SP). In imaging mode, back-scattered SHG signal (blue) is reflected by the dichroic mirror (DC) and detected in the non-descanned path through a bandpass emission filter (BP, FF01-445/20, Semrock) by a hybrid detector (HyD) from Leica. During calibration, the OL is removed and a polarization analyzer consisting of a pinhole (PIN), polarizer (POL), and photodiode (PD) is inserted.

**Table 1:** Compensation module main components.

Component	Quantity	Model	Vendor
Achromatic quarter-waveplate	1	AQWP10M-980	Thorlabs
Achromatic half-waveplate	1	AHWP10M-980	Thorlabs
Motorized rotation stage with controller	2	KPRM1E/M	Thorlabs

The polarization analyzer is also based on the method proposed by Romijn et al. [11], although with substantially improved sampling rate and acquisition speed. Our system comprises four key components (Table 2): a motorized rotation stage capable of 5 Hz (1 800°/s) rotation speed, a polarizer mounted on the rotation stage, a photodiode for measuring transmitted power, and a 16-bit data acquisition (DAQ) device selected for increased dynamic range compared to lower bit-depth alternatives, which maximizes ellipticity measurement sensitivity. The mechanical assembly is illustrated in S2 Fig and computer-aided design (CAD) files are available in S1 File (SolidWorks Premium 2022 SP5.0, Dassault Systèmes).

**Table 2:** Polarization analyzer main components.

Component	Model	Vendor
Pinhole	P4000K1	Thorlabs
Polarizer	LPVIS050-MP2	Thorlabs
Motorized rotation stage	DDR25/M and KBD101	Thorlabs
Photodiode	SM1PD1A	Thorlabs
Bias module	PBM42	Thorlabs
Variable terminator	VT2	Thorlabs
Data acquisition device	USB-6002	NI

The rotation stage completes individual turns in approximately 350 ms, including acceleration and deceleration phases. The stage controller outputs digital triggers at every degree from 0° to 359°. Since the DAQ does not support retriggerable tasks (hardware-triggered finite acquisition that resets on each trigger), data are acquired continuously from both the photodiode and the trigger signal simultaneously. Acquisition runs for 360 ms at 25 000 samples/s per channel, yielding 9 000 samples per channel. This rate is limited by the DAQ’s maximum aggregate sampling rate of 50 000 samples/s shared between channels. Post-processing identifies trigger positions in the recorded trigger channel to reconstruct the photodiode signal versus polarizer rotation angle. To reduce measurement variation, the sample at each trigger position is averaged with its two subsequent samples (angular displacement calculated in S1 Appendix). The complete electronics and wiring connections are shown in S3 Fig.

The DAQ analog input exhibits an offset that stabilizes after a 20 min warm-up period. This offset is recorded by averaging the channel readout for 6 s in complete darkness and typically ranges from –2 mV to –3 mV in our measurements. Both raw analog data and offset values are saved; background subtraction is performed during post-processing alongside trigger-based resampling. Operating parameters require careful consideration: photodiode linearity is verified by confirming proportional response to laser power variations, and the load resistance is maintained below 5 kΩ to ensure adequate bandwidth for the 25 ksamples s^–1^ acquisition rate. Detailed bandwidth calculations are provided in S2 Appendix. All measurements in this work employed a 500 Ω load with 10 V bias voltage, constrained by DAQ analog output specifications. Validation against a power meter demonstrated agreement for ellipticity values above approximately 0.074 ellipticity (see S4 Fig). This comparison also serves as a characterization of the measurement noise floor, since the agreement between the two independent detectors bounds the measurement uncertainty at each ellipticity level.

For comparison, a single ellipticity measurement in Romijn et al. [11] required approximately 17 s. While single measurement duration is not documented in this previous work, an analysis of the published code reveals 6 samples at 30° intervals (line 116 of polarization_map.m) over 0° to 150° (line 296 of polarization_map.m), with 1 s required for each power measurement and 25°/s maximum rotation speed. Assuming a 25°/s^2^ acceleration with a trapezoidal velocity profile yields an approximately 2.2 s travel time between samples. In contrast, our polarization analyzer completes a measurement in 360 ms, which constitutes a substantial improvement although the overall calibration time remains limited by the waveplate rotation stages (see Results and discussion).

### Software implementation

We provide the complete Python codebase for experimental data acquisition and analysis in S2 File (https://github.com/Omnistic/residual_ellipticity_in_pshg_hardware_and_analysis). The core computational routine implements the intensity fit for single polarization measurements following Eq 7 from Romijn et al. [11]. We use SciPy’s curve_fit function [13] with several modifications to improve numerical stability. The photodiode signal is scaled by a factor of 10 000 to avoid small numerical values from the DAQ (resolution is 0.305 mV). To enforce the physical constraint $E_{max}\geq E_{min}$, we reparameterize using an intermediate variable *k* where $E_{max}=E_{min}+k^{2}$, fitting the parameters $\left(\alpha_{max},E_{min},k\right)$ without requiring initial guesses. The bounds are set to $\left(0,\sqrt{100000}\right)$ for $\left(E_{min},k\right)$ based on the 10 V maximum analog input range, and (0,π) for $\alpha_{max}$ in radians.

For the ellipticity map fitting (Eq 1–3 in Romijn et al. [11]), we apply the same photodiode signal scaling factor. The bound for $I_{0}={E_{0}}^{2}$ is set to (0,100000). Note that *I*_0_ is missing from Eq 3 in Romijn et al. [11] but appears as *E*_0_ in their Eq 1 and as *I*_0_ in their code implementation (line 772 of polarization_map.m). The reflection ratio parameter γ only has a positivity constraint using the bounds (0,inf) and all the angles are constrained within (−π,π) in radians. A generic initial guess is provided as 10 000 for *I*_0_ (actual photodiode signal voltage on the order of 1 V), 1.0 for γ, and 0° for all the angles. The optimal compensation curves that minimize ellipticity (Eq 4 in Romijn et al. [11]) are determined using SciPy’s root function. To ensure convergence to the correct solutions, the user supplies two initial QWP angles that minimize ellipticity on average across all HWP angles, identified by visual inspection of the ellipticity map.

## Results and discussion

To establish baseline performance, we first measured ellipticity as a function of relative polarization angle ($\alpha_{max}$) using only the HWP, without the QWP. Measurements were taken at both the entrance port (right after the HWP) and the sample plane (Fig 2A). All sample-plane measurements were performed at the position where the sample would normally be located, with the objective lens removed, as in the calibration procedures of Romijn et al. [11] and Chou et al. [9]. As a result, the measurements characterize the polarization state delivered to the objective and do not include objective-induced effects. At the entrance port, the ellipticity remains below 0.05. There is variation of the ellipticity with polarization angle, but as it falls within the laser polarization specification (given as an extinction ratio 500:1, corresponding to an ellipticity of $\sqrt{\frac{1}{500}}=0.045$) and near or below the reliability threshold of our photodiode-based analyzer (S4 Fig), where the photodiode may modestly underestimate ellipticity, we consider the polarization to be effectively linear. By comparison, at the sample plane, the ellipticity shows periodic variation with two peaks and two valleys, ranging from 0.06 to 0.25.

**Fig 2:**
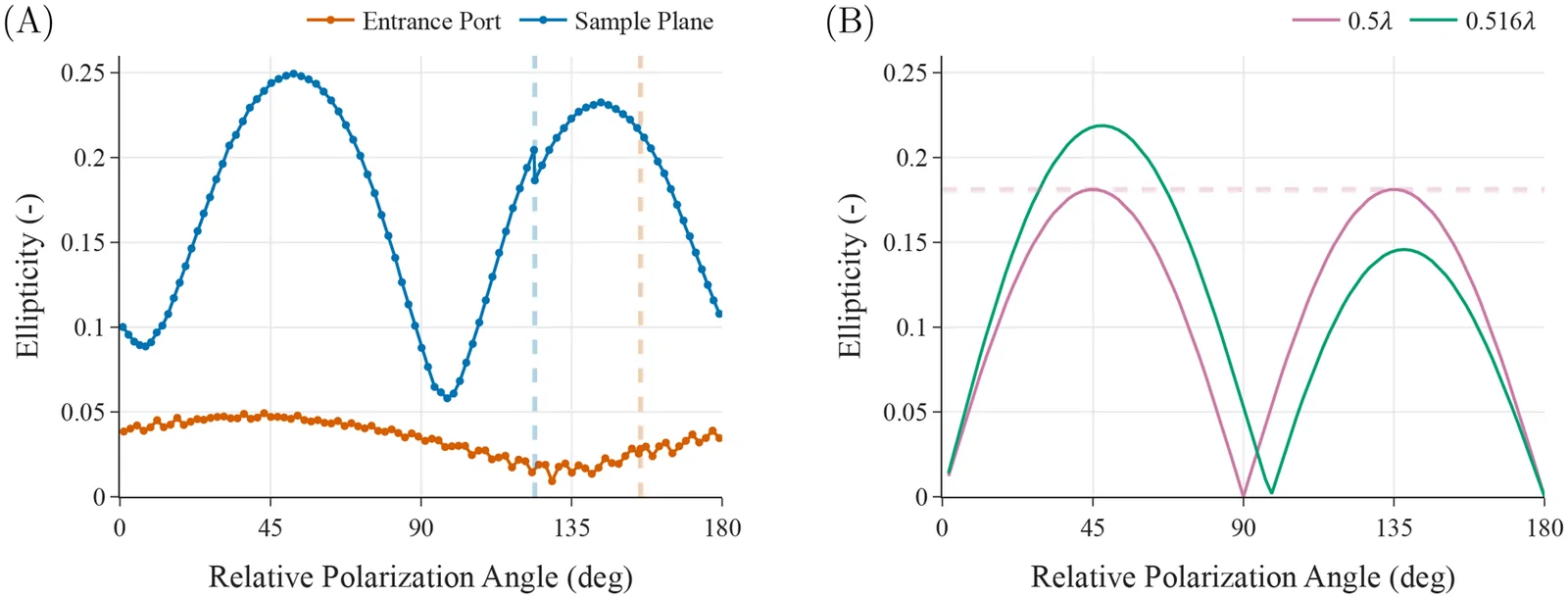
Preliminary ellipticity measurements and simulation. (A) Measured ellipticity as a function of relative polarization angle ($\alpha_{max}$) using only the HWP, without the QWP. Measurements were taken at the entrance port (right after the HWP, orange) and at the sample plane (blue). At the entrance port, ellipticity remains below 0.05, within laser specification. At the sample plane, ellipticity shows periodic variation with two peaks and two valleys, ranging from 0.06 to 0.25. Vertical dashed lines indicate the HWP motor zero position for each measurement. (B) Simulated ellipticity using a Jones matrix model in OpticStudio 2026 R1 (Ansys Zemax). The optical path consists of the QWP and HWP (modeled as linear retarders, with the QWP retardance fixed at zero throughout this simulation), followed by five silver-protected mirrors at 45° based on Chou et al. [9], and a dichroic mirror modeled as a linear retarder with 12.1° retardance. Simulation with ideal HWP (retardance = 0.5λ, pink curve) produces symmetric peaks and valleys, while simulation with manufacturer-specified HWP (retardance = 0.516λ, green curve) reproduces the asymmetry observed in the sample plane data (panel A), where peaks reach different heights and valleys reach different minima. A pink horizontal dashed line marks the first peak maximum to highlight this asymmetry.

To understand the ellipticity variation pattern at the sample plane, we consider the differential reflection properties of metallic mirrors at non-normal incidence. In our optical system, mirrors are generally positioned at 45° to the beam path. At each reflection, the beam’s polarization can be decomposed into *s*-polarized (perpendicular to the plane of incidence) and *p*-polarized (parallel to the plane of incidence) components, which experience different phase shifts upon reflection from the metallic surface (see S5 Fig). When the beam polarization is oriented as purely *s* or purely *p*, no ellipticity is introduced, giving rise to the two valleys in the pattern separated by 90° (*s* and *p* polarizations are orthogonal). The maximum ellipticity (peaks) occurs at intermediate angles, specifically halfway between the *s* and *p* orientations. Rotating the HWP from 0° to 90° sweeps through these different polarization orientations (0° to 180°), producing the periodic pattern. While this explains the basic two-peak, two-valley structure, additional optical components—including the dichroic mirror and potentially other elements in the commercial microscope system—add further complexity. To validate our understanding, we performed a Jones matrix [14] simulation based on the optical model from Chou et al. [9] (illustrated in their figure 1), which consists of a cascade of five mirrors followed by a dichroic mirror. We borrowed this mirror cascade model from Chou et al. only for the fixed optics between the compensation module and the sample, while the waveplate ordering itself followed our experimental configuration with the QWP positioned nearest the laser and the HWP nearest the sample as in Romijn et al. [11] (rather than the HWP-QWP ordering illustrated in Chou et al.’s figure 1). We refer to Materials and methods for the practical implications of this ordering choice. We modeled the mirrors as silver-protected reflectors, a reasonable assumption given typical near-IR mirror coatings, as the specific coatings in our commercial system are not documented. We further modeled the dichroic mirror as a linear retarder with 12.1° retardance, the value determined by the Romijn et al. [11] calibration method applied to our system (see below), assuming the fitted system retardance is attributable entirely to the dichroic mirror. The simulation was implemented in OpticStudio 2026 R1 (Ansys Zemax), where the HWP angle was swept programmatically using the ZOS-API via the open-source ZOSPy Python wrapper [15]. At each HWP angle, we calculated the output polarization state at the sample plane. Linear retarders (including the waveplates and dichroic mirror) were modeled as Jones matrix surfaces with the form:


$$\begin{array}{c} J(\Gamma ,\beta )=(\begin{array}{cc} A_{r}+iA_{i} & iB_{i}\\ iB_{i} & A_{r}-iA_{i} \end{array}), \end{array}$$ (1)

where


$$\begin{array}{cc} A_{r} & =\cos\left(\frac{\Gamma }{2}\right),\\ A_{i} & =\sin\left(\frac{\Gamma }{2}\right)\cos(2\beta ),\\ B_{i} & =\sin\left(\frac{\Gamma }{2}\right)\sin(2\beta ), \end{array}$$ (2)

with Γ the retardance and β the fast axis angle of the retarder. To simplify parameter testing, we implemented these matrix elements using custom ZPL macro solves (one for each coefficient in Eq 2). The ZPL macro implementation is detailed in S3 Appendix, and OpticStudio files and simulation codes are provided in S3 File (https://github.com/Omnistic/residual_ellipticity_in_pshg_simulations). Ellipticity and polarization angle were extracted from the simulated intensity as described in S5 Appendix. Fig 2B shows the simulated ellipticity pattern as a function of the relative polarization angle. With an ideal half-waveplate (retardance = 0.5λ, where λ is the wavelength), the simulation produces a periodic pattern ranging from 0.0–0.18 with symmetric peaks and valleys. However, using the manufacturer-specified retardance of 0.516λ at 880 nm introduces asymmetry: the two peaks reach different heights and the second valley shifts from 90° to 98°. This asymmetry matches the behavior observed in our experimental data.

We then experimentally applied the full compensation method of Romijn et al. [11], measuring ellipticity as a function of both QWP and HWP angles to create two-dimensional ellipticity maps at the entrance port and sample plane (the latter with the objective lens removed, Fig 3A). Our improved acquisition speed enabled maps with double the angular sampling resolution while reducing acquisition time from approximately 2 h to 30 min per map compared to the original implementation. To confirm that the polarization state entering the microscope is stable over this acquisition time, we measured the ellipticity of the input beam (at the entrance port) at a fixed waveplate configuration chosen to yield an ellipticity of approximately 0.5 (where our measurement method is accurate and consistent, see S4 Fig), once per minute for two hours. We observed that it remained stable throughout (mean 0.4943, standard deviation 0.0010; S6 Fig), well beyond the duration of a single map acquisition.

**Fig 3:**
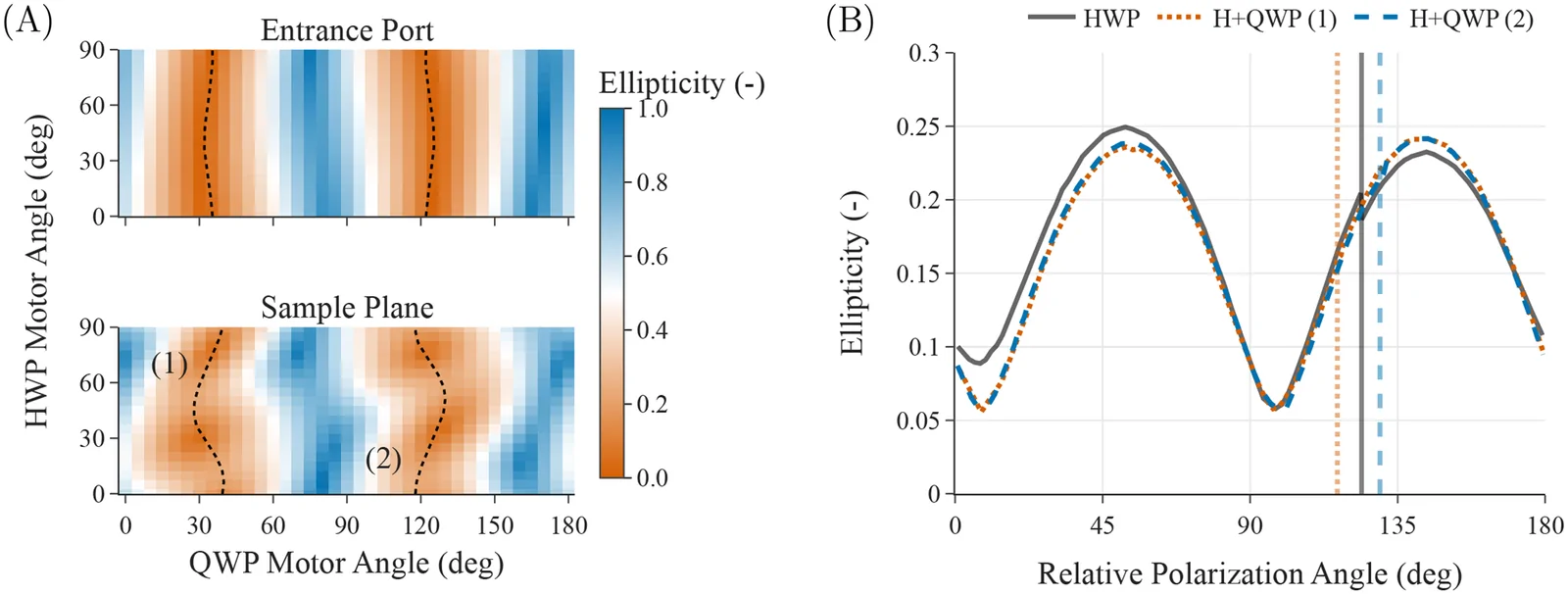
Two-dimensional ellipticity maps and compensation performance. (A) Ellipticity as a function of QWP and HWP angles at the entrance port (top) and sample plane (bottom), measured following Romijn et al. [11] with double the angular sampling resolution. Each map was acquired in approximately 30 min. At the entrance port, the map displays nearly vertical stripes indicating minimal ellipticity requiring small QWP corrections. At the sample plane, the pattern exhibits pronounced waviness with substantially larger QWP amplitude required for compensation. Dashed black lines indicate the two optimal compensation curves for achieving linear polarization at each HWP angle, calculated using Eq 4 from Romijn et al. [11] and labeled (1) and (2) on the sample-plane map. Even along these optimal curves, ellipticity varies substantially, reaching values up to 0.24 in certain regions. (B) Ellipticity measured at the sample plane along the two optimal compensation curves, extracted from the map in panel A (bottom) and resampled at 1° intervals. The HWP-only baseline (black, solid) is shown alongside the two compensation solutions, H + QWP (1) (orange, dotted) and H + QWP (2) (blue, dashed), which correspond to curves (1) and (2) in panel A. The two compensation solutions overlap closely. While compensation produces more symmetric curves than the HWP-only baseline, substantial residual ellipticity persists as a function of HWP angle. The zero positions of the two compensation solutions are offset by approximately 13° (vertical lines, shown in the corresponding line style).

At the entrance port, the ellipticity map displays a pattern of nearly vertical stripes, indicating minimal ellipticity that requires only small QWP rotations for compensation. This is consistent with the single-HWP measurements (Fig 2A), confirming that the beam remains nearly linearly polarized at this location. In contrast, the sample plane map exhibits substantial distortion: the pattern develops pronounced horizontal waviness, and the amplitude of QWP rotation required for compensation increases significantly. This reflects the substantial ellipticity introduced by the optical path between the entrance port and sample plane (objective lens excluded, as per Romijn et al. [11]; see Materials and methods).

Fitting the ellipticity maps to the model of Romijn et al. (Eq 1–3) provides estimates of key system parameters (Table 3). In our first experiment (Exp 1), the fitted system retardance δ increased substantially from the entrance port to the sample plane (3.4° to 12.1°), confirming substantial birefringence accumulation through the microscope optics. The fitted QWP orientation angle $\phi_{0}$ remained nearly identical between the two locations, as expected since the QWP mounting was not changed, whereas the fitted HWP angle $\theta_{0}$ and polarization analyzer angle $\alpha_{0}$ both differed noticeably between the entrance port and the sample plane. The latter may partly reflect the different mounting orientation required for the polarization analyzer at the sample plane due to space constraints. To assess whether this discrepancy was reproducible, we repeated the full map acquisition and fitting procedure on two additional, separate days (Exp 2 and 3), including three separate map acquisitions at the sample plane within a single session (Exp 3, Sample 1–3). As the instrument is shared with other users, the waveplates were removed from their motorized mounts and reinstalled between experiments. Across all three experiments, $\phi_{0}$ remained remarkably stable, while the same $\theta_{0}$ and $\alpha_{0}$ discrepancy between locations persisted, with some additional spread between experiments.

**Table 3:** Fitted parameters from ellipticity maps. Parameters obtained by fitting ellipticity maps to the model of Romijn et al. (Eq 1–3). *I*_0_ represents the reference intensity, γ is the reflection ratio parameter, δ is the cumulative system retardance, and $\theta_{0}$, $\phi_{0}$, and $\alpha_{0}$ represent the angular offsets of the HWP fast axis, QWP fast axis, and polarization analyzer transmission axis, respectively, relative to their motor zero positions. Exp 1, 2, and 3 correspond to three independent map acquisitions performed on separate days. Due to the instrument being shared with other users, the waveplates were removed from their motorized mounts and reinstalled between experiments, and absolute alignment was not necessarily identical from one experiment to the next. Due to space constraints, the polarization analyzer had to be mounted at a different angle at the sample plane than at the entrance port, which partially accounts for the offset in $\alpha_{0}$ between these two locations. Exp 3 includes three separate ellipticity map acquisitions at the sample plane, taken back-to-back within a single session (Sample 1–3), to assess reproducibility across repeated acquisitions at that location. Together with the entrance-port input-beam stability check over two hours (S6 Fig), these results argues against a systematic drift in the polarization state during map acquisition. †Entrance and sample measurements shown in the main text (Fig 3A).

		*I*_0_	γ	δ (°)	$\theta_{0}$ (°)	$\phi_{0}$ (°)	$\alpha_{0}$ (°)
Exp 1†	Entrance	2.01	0.99	3.40	18.07	33.69	11.28
	Sample	1.96	1.07	12.10	25.61	33.85	−6.00
Exp 2	Entrance	2.79	0.99	3.51	18.96	32.69	13.06
	Sample	2.15	1.07	13.92	26.43	32.93	−4.95
Exp 3	Entrance	1.84	1.00	2.97	18.21	32.90	11.57
	Sample 1	1.60	1.07	11.21	26.09	33.03	−5.27
	Sample 2	1.61	1.07	11.09	26.01	33.01	−5.43
	Sample 3	1.62	1.07	11.01	26.00	33.01	−5.45

As with the HWP-only simulation (Fig 2B), accounting for the real, manufacturer-specified waveplate retardance rather than assuming ideal retardance again proved important: it appears to explain the $\theta_{0}$ and $\alpha_{0}$ discrepancy observed between the entrance port and sample plane, since the Romijn et al. model assumes ideal waveplate retardance and does not account for this deviation. To test this, we used the Jones matrix simulation described above to generate synthetic ellipticity maps with known ground-truth waveplate and analyzer angles, and fitted them using our procedure, comparing ideal and manufacturer-specified waveplate retardances at system retardances δ comparable to our experimental entrance-port and sample-plane values (S1 Table). Fits were performed multiple times per condition and constrained to avoid convergence to degenerate solutions. This confirmed that $\phi_{0}$ can be robustly recovered regardless of waveplate retardance, whereas introducing realistic retardance substantially degrades the accuracy of $\theta_{0}$ and $\alpha_{0}$. This pattern led us to examine the δ=0 limit analytically, where we found that $\theta_{0}$ and $\alpha_{0}$ are not individually identifiable, while $\phi_{0}$ remains identifiable (S4 Appendix). The larger error observed at the lower system retardance (entrance port) compared to the higher one (sample plane) is consistent with this degeneracy, though we have not characterized how the error scales with δ away from this limiting case.

Despite these limitations in individually resolving $\theta_{0}$ and $\alpha_{0}$, we used the fitted model to identify waveplate settings expected to compensate for the ellipticity of the system, following the approach of Romijn et al. The optimal compensation curves (curves (1) and (2) in Fig 3A), representing the two solutions for achieving linear polarization at each HWP angle, reveal a critical limitation: even where QWP and HWP angles are optimally adjusted, ellipticity varies substantially along these curves, reaching values up to approximately 0.24 at certain HWP angles while dropping near zero at others. To quantify this effect, we extracted these compensation curves from the ellipticity maps, resampled them at 1° intervals, and measured the resulting ellipticity at the sample plane (Fig 3B). While compensation produces more symmetric curves compared to the HWP-only case, substantial residual ellipticity persists and varies significantly as a function of HWP angle. The two compensation solutions overlap closely, with their zero positions offset by approximately 13° (indicated by vertical lines in the corresponding line style). Both this shift and the residual ellipticity variation were already present in Romijn et al.’s data (their figure 6b and 6d), although not extensively discussed.

Because ellipticity varies with polarization angle, it could in principle affect the shape of polarization-dependent SHG intensity polar plots, which are commonly used to infer sample orientation and microstructural organization. As a quantitative assessment of this effect on downstream analysis is beyond the scope of the present study, we restricted our observations to the polarization state itself. Our key finding is that the waveplate compensation approach provided minimal improvement in our system: while ellipticity varied from 0.06 to 0.25 with HWP only, the residual ellipticity after full compensation remained comparable, simply with improved symmetry. This suggests that, for systems with relatively low retardance to compensate as in our case, the practical benefit of QWP compensation is limited.

We hypothesized that this residual ellipticity arises from the combination of wavelength-dependent dichroic mirror birefringence and the broad spectral bandwidth of femtosecond laser pulses. Dichroic mirror birefringence is well documented in the literature [12], including its strong wavelength dependence. Schön et al. measured dichroic mirror retardance using ellipsometry, demonstrating variations exceeding 50° over spectral ranges of 10 nm to 20 nm (their figure 6b).

This wavelength dependence becomes particularly relevant for polarization-resolved SHG microscopy because femtosecond laser sources possess relatively large spectral bandwidths. Our Spectra-Physics Mai Tai laser at 880 nm has a specified bandwidth of 12.5 nm (FWHM), comparable to the spectral range over which dichroic retardance can vary by tens of degrees. While a single retardance value can be compensated by appropriate QWP and HWP settings, each wavelength component within the pulse bandwidth experiences different retardance from the dichroic mirror. The waveplates, however, can only be optimized for a single wavelength or narrow spectral range. Consequently, wavelength components away from the optimization point would accumulate different amounts of ellipticity, which may result in residual ellipticity that cannot be fully eliminated by a fixed waveplate configuration.

To explore this hypothesis, we extended our previous Jones matrix simulations to incorporate the polychromatic nature of femtosecond pulses. We modeled the laser spectral bandwidth as 15 discrete wavelengths, spanning the range where the Gaussian weight distribution (FWHM = 12.5 nm, centered at 880 nm) falls to 1/*e*^2^ of its peak value. This number of wavelengths was chosen based on a convergence analysis showing it closely approximates the result obtained with a substantially finer wavelength sampling (S7 Fig). The dichroic mirror retardance was modeled to vary linearly from –17.9° to 42.1° across this bandwidth, centered at 12.1° at 880 nm, spanning a total range of 60° consistent with the measurements of Schön et al. [12]. The waveplates’ own retardance was held fixed across wavelength in this simulation, as their achromatic design (Table 1) makes this wavelength dependence negligible over the bandwidth considered here. For each QWP and HWP angle combination, we calculated the transmitted intensity as a function of polarization analyzer angle for all 15 wavelengths independently, then summed these intensity curves incoherently to simulate the detector response. Ellipticity was then extracted from the summed intensity curve using the same fitting procedure as for experimental data (ellipticity extraction details in S5 Appendix). We provide the simulation code and OpticStudio files in S3 File.

Fig 4 brings together this polychromatic simulation with our earlier monochromatic one, comparing all four combinations of waveplate retardance (ideal or real) and illumination bandwidth (monochromatic or polychromatic), and contrasting the compensation curve obtained by fitting the model of Romijn et al. with the curve obtained by directly taking the minimum ellipticity from each map at every HWP angle. Maps were computed with the HWP and QWP each sampled at 1° intervals, and the polarizer sampled at 8° intervals (see S5 Appendix). For monochromatic illumination with ideal waveplate retardances (Fig 4A and 4B), the curve obtained by fitting the model of Romijn et al. remains essentially flat, on the order of 10^–5^, while the minimum-ellipticity curve shows some noise but does not exceed 0.01. The beam is therefore essentially linearly polarized across the full range of polarization angles, with the small residual noise in the minimum-ellipticity curve likely reflecting the discretization of the map rather than a real effect. Introducing real waveplate retardances alone (monochromatic case, Fig 4C and 4D) reveals a modest but consistent effect: the minimum-ellipticity curve remains below 0.01, aside from the same discretization noise, whereas the curve obtained by fitting the model of Romijn et al. already shows a clear pattern of two peaks and two valleys. The peaks and valleys reach a maximum of approximately 0.05 and a minimum of approximately 0.017 respectively and never return to zero, which is likely related to the errors that real waveplate retardance introduces in the fitted $\theta_{0}$ and $\alpha_{0}$ (S1 Table and S4 Appendix), although we have not directly verified this link. Incorporating spectral bandwidth has a larger effect. For the polychromatic simulations (Fig 4E–4H), we used 15 wavelengths and a dichroic retardance span of ±30° around 12.1°. With ideal waveplate retardances (Fig 4E and 4F), the two curves remain close to one another, as expected since the waveplates are ideal. Additionally, both now show a pattern of two peaks and two valleys, approaching zero at the valleys (with some noise in the minimum-ellipticity curve) and reaching a maximum of approximately 0.1. The region near the arrowhead (HWP ≈ 60°, QWP ≈ 166°), which showed near-zero ellipticity in the monochromatic case, becomes blurred, with a residual ellipticity pattern reappearing. Combining real waveplate retardances with polychromatic illumination (Fig 4G and 4H) reproduces both effects together: the minimum-ellipticity curve approaches zero at its valleys with a maximum of approximately 0.1, while the curve obtained by fitting the model of Romijn et al. never returns to zero, with a minimum of approximately 0.017 and a maximum of approximately 0.11. Overall, real waveplate retardance therefore introduces a comparatively modest error in identifying the optimal compensation angles, whereas spectral bandwidth introduces a larger effect that compensation based on a single wavelength cannot capture.

**Fig 4:**
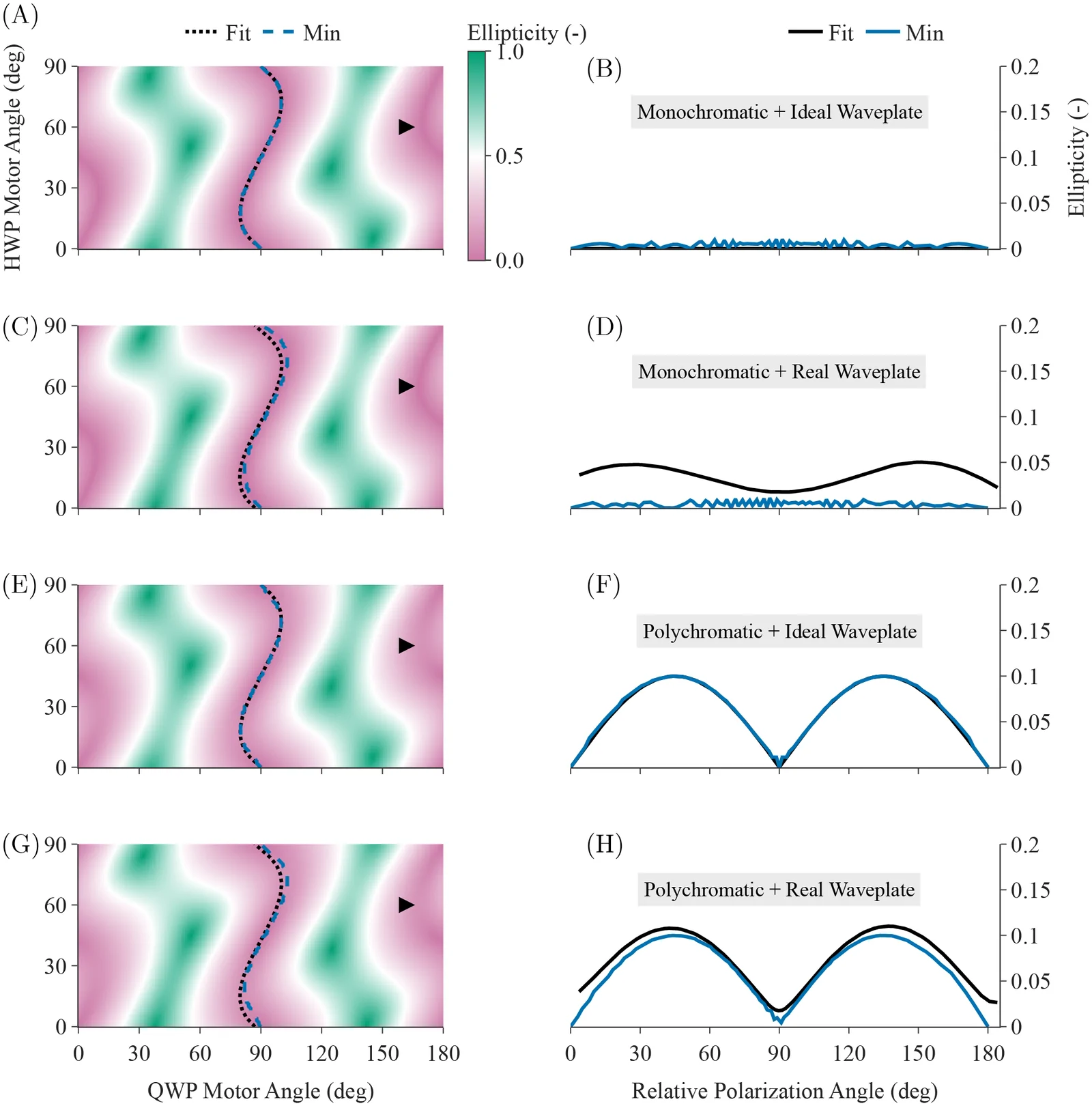
Effect of waveplate retardance and spectral bandwidth on ellipticity compensation. Simulated ellipticity maps (A, C, E, G) and corresponding ellipticity as a function of relative polarization angle (B, D, F, H) for four conditions: monochromatic simulation with ideal waveplate retardances (A-B); monochromatic simulation with manufacturer-specified real waveplate retardances (C-D); polychromatic simulation with ideal waveplate retardances (E-F); and polychromatic simulation with real waveplate retardances (G-H). For the QWP-near-90° solution, each map (A, C, E, G) shows two overlaid paths: the linear-polarization solution obtained by fitting the model of Romijn et al. (dotted black), and the path obtained by taking, at each HWP angle, the QWP angle giving the minimum ellipticity directly from the map (dashed blue). The corresponding ellipticity along these two paths is shown in panels B, D, F, and H (solid black and solid blue lines, respectively). Arrowheads in all four maps mark the same representative location (QWP angle ≈ 166°, HWP angle ≈ 60°) on the other solution branch. This branch forms a continuous path of near-zero ellipticity in the monochromatic case (A, C), but becomes blurred in the polychromatic case (E, G), with residual ellipticity reappearing as a pattern of two peaks and two valleys, qualitatively similar to the residual ellipticity observed experimentally at the sample plane (Fig 3B).

While our simulations do not quantitatively match the experimental amplitude (which reaches approximately 0.24), they support the hypothesis that wavelength-dependent dichroic retardance combined with femtosecond pulse bandwidth may contribute to the residual ellipticity observed under waveplate-based compensation. To assess how sensitive this residual ellipticity is to our modeling assumptions, we repeated the polychromatic simulation (real waveplate retardances, 12.5 nm bandwidth) while varying the total span of the dichroic retardance around the central value of 12.1°, from ±0 (i.e., the same retardance at every wavelength) up to ±60° in steps of 10° (S8 Fig). We only observed the simulated ellipticity amplitude to exceed 0.2, reaching approximately 0.22 and approaching but not fully reproducing the experimental maximum of approximately 0.24, at the most extreme span tested (±60°). Smaller, more conservative spans produced substantially lower amplitudes. We are reluctant to consider a ±60° span (120° total) over a 12.5 nm bandwidth representative of our system, since the retardance spectrum reported by Schön et al. [12] is of limited resolution and our own dichroic mirror is likely to differ substantially from theirs, making their measurements an unreliable estimate of our specific mirror’s retardance slope. Separately, we also varied the illumination bandwidth (monochromatic, 2 nm, 12.5 nm, and 25 nm) while keeping the dichroic retardance-versus-wavelength slope fixed at the value used for our 12.5 nm reference condition (span of ±30° around 12.1° over that bandwidth; S9 Fig). The 2 nm bandwidth was chosen as roughly representative of a picosecond laser source, which would generate substantially less SHG signal than a femtosecond source of comparable average power, but would be expected to reduce the polychromatic contribution to residual ellipticity. Under this fixed slope, the 2 nm bandwidth samples only a small central portion of the retardance range, while the 25 nm bandwidth extrapolates the same slope further, to a larger effective span. As expected, smaller bandwidth produced lower residual ellipticity.

The discrepancy in amplitude between our experimental measurements and computational simulations could arise from several factors: our simplified assumption of a linear dependence between dichroic retardance and wavelength, uncertainty in the actual dichroic retardance spectrum of our specific microscope, or contributions from other wavelength-dependent optical elements. Unfortunately, due to the closed, commercial nature of our system, the exact optical train inside the microscope is not fully documented by the manufacturer, and it is possible that more than one dichroic mirror, or other wavelength-dependent components, lie along the beam path between the entrance port and the sample plane, each potentially contributing additional, unaccounted-for retardance. Direct measurement of our dichroic mirror retardance spectrum would be required to confirm this mechanism and represents an important direction for future work. We were unfortunately unable to perform it here as the dichroic mirror was used continuously in a shared facility instrument and could not be removed for ellipsometry.

## Conclusions

In this work, we have implemented and characterized the waveplate-based polarization compensation method previously proposed by Romijn et al. [11] on a commercial Leica TCS SP8 MP multiphoton microscope, and introduced modifications to achieve a substantially improved acquisition speed enabling high-resolution ellipticity mapping as a function of the QWP and HWP rotation angles. Our systematic measurements confirm that the compensation approach successfully symmetrizes angle-dependent ellipticity patterns. However, substantial residual ellipticity persists even under optimal waveplate configurations, varying periodically as a function of output polarization angle with amplitudes up to 0.24. We also noted that the fitted half-waveplate and polarization analyzer angles differed between the entrance port and sample plane. Simulations attributed this discrepancy to the real, manufacturer-specified waveplate retardance, which the model of Romijn et al. does not account for. A simplified analytical treatment further suggests that these two parameters may become difficult to separate at low system retardance, offering a possible, though not fully verified, explanation for such fitting errors.

Through Jones matrix simulations, we found that accounting for real, manufacturer-specified waveplate retardance had a comparatively modest effect on the resulting compensation curves, which is consistent with the parameter degeneracy noted above. Incorporating the polychromatic nature of femtosecond pulses had a substantially larger effect: even with ideal waveplates, spectral bandwidth alone reintroduced residual ellipticity that ideal, monochromatic compensation would not predict. While each wavelength component can theoretically be compensated, fixed waveplates can only optimize for a single wavelength, thus leaving other spectral components with residual ellipticity. This suggests that the effect may be a limitation of waveplate-based compensation for femtosecond laser systems rather than solely an implementation issue. Our simulations reproduced this residual ellipticity only qualitatively, as matching the full experimental amplitude required an unusually large span of dichroic retardance across the illumination bandwidth, which we consider unlikely to be representative of our specific, undocumented commercial optical system. The combination of wavelength-dependent dichroic mirror birefringence and femtosecond pulse bandwidth therefore remains, at this stage, a plausible but unconfirmed contributing mechanism, pending direct ellipsometric characterization of the dichroic mirror itself. It should also be noted that our microscope exhibited relatively modest system retardance (12.1° at the sample plane). Systems with substantially higher birefringence may see more dramatic improvement from waveplate compensation, even though the residual ellipticity variation we observed would still be expected to persist. In our case, the initially low ellipticity meant that the compensation approach offered limited practical benefit. While the specific magnitude of residual ellipticity we report is, in principle, a property of our particular instrument, we note that a comparable magnitude and a similar periodic pattern are also present, though not extensively discussed, in the originally published data of Romijn et al. (their figure 6b), which was obtained on the same class of commercial instrument as ours. This suggests that the magnitude of the effect may be more consistent across similar systems than would be expected from an idiosyncratic property of a single instrument. We however do not have data from other microscope models to test this further. More broadly, the underlying physical mechanisms we investigate (differential reflection phase shifts at metallic mirrors, and wavelength-dependent retardance in dichroic mirror coatings) are general properties of the optical elements involved, and are documented independently of our specific instrument [12]. We would thus expect the qualitative limitation we describe here to be relevant to other waveplate-compensated femtosecond multiphoton systems, though its practical significance would depend on the magnitude of a given system’s wavelength-dependent retardance relative to its spectral bandwidth. Since, as previously mentioned, all of our measurements were performed at the sample plane with the objective removed following the original methods of Romijn et al. and Chou et al., the present study characterizes the polarization state delivered to the objective and does not address objective-induced depolarization [8]. The latter would be a further consideration for full-system polarization accuracy in high-NA imaging.

These findings have important implications for quantitative polarization-resolved SHG microscopy. For applications requiring precise polarization control, we suggest two potential approaches for future investigation. First, specimen rotation: rather than attempting to compensate the beam polarization across all angles through the microscope optics, one could minimize ellipticity at the sample plane (with a QWP) for a single beam polarization orientation and instead rotate the specimen. While this approach introduces technical challenges in maintaining the field of view relative to the rotation axis, necessitating motorized x-y translation and rotation stages, it would circumvent the wavelength-dependent retardance problem entirely. Second, picosecond laser sources with significantly narrower spectral bandwidths could reduce the wavelength-dependent effects we have identified. While the longer pulse duration would reduce SHG generation efficiency, the improved polarization control may benefit quantitative applications where signal-to-noise is less limiting than polarization accuracy. Additionally, if feasible, achromatic dichroic mirrors designed for minimal retardance variation across the 10–20 nm bandwidth of femtosecond laser pulses at the operating wavelength could address the wavelength-dependent limitations identified here.

## Supporting information

S1 Fig Compensation module installation. Photograph of the compensation module showing motorized rotation stages for the quarter-waveplate (QWP, orange arrow) and half-waveplate (HWP, blue arrow) positioned in the beam path before the microscope entrance port. The polarization analyzer is shown mounted immediately after the compensation module for entrance port measurements; the same analyzer was also positioned at the sample plane for sample plane measurements (not shown), with the objective lens removed as in Romijn et al. [11]. Enclosures are open for the photograph; all components are enclosed during measurements for laser safety.
(TIF)

S2 Fig Mechanical assembly of the polarization analyzer. CAD assembly of the polarization analyzer. Arrows indicate component positions with corresponding Thorlabs model numbers labeled. Component models were obtained from Thorlabs. The assembly shows the photodiode detector (SM1PD1A) mounted in the optical path; a power meter sensor could be interchanged using a quick mount (SM1QA) for validation measurements. Complete SolidWorks assembly files are provided in the supplementary materials archive. Electronics, wiring, and data acquisition connections are shown in S3 Fig.
(TIF)

S3 FigElectronics and control system schematic.Wiring diagram showing the photodiode detector (SM1PD1A) with bias module (PBM42) and variable terminator (VT2), data acquisition device (USB-6002), and motor control architecture. The photodiode operates with 10 V bias and 500 Ω load. The DAQ provides the DC bias voltage to the PBM42 via analog output AO0, limited to 10 V but sufficient for this application. Motor controllers (KBD101 for analyzer rotation; 2× KDC101 for waveplates) connect via KEH3 hub for single USB interface. The KBD101 digital trigger output serves two purposes: it initiates the DAQ acquisition task (DI0) and is simultaneously recorded as an analog input (AI0) alongside the photodiode signal (AI1). This dual connection compensates for the DAQ’s lack of retriggerable task support, enabling precise synchronization of photodiode measurements with angular position (see Materials and methods).(TIF)

S4 Fig Validation of photodiode against power meter. Comparison of ellipticity measurements using the photodiode detector (SM1PD1A, orange) and a power meter (Thorlabs PM100D with S121C sensor, blue) across a range of ellipticity values. The S121C sensor is also photodiode-based with similar specifications to the SM1PD1A, enabling direct comparison. Both detectors feature large photosensitive areas, providing tolerance to alignment variations. To validate the photodiode across different ellipticity levels, a single QWP (without HWP) was positioned in the beam path at various fast axis angles relative to the beam polarization to deliberately introduce controlled amounts of ellipticity. Each panel shows boxplots of repeated measurements: 20 measurements per detector without the QWP (NO QWP), and 10 measurements per detector with the QWP at fast axis angles of 0°, 5°, 10°, 15°, 30°, and 45°. The y-axis of each panel is centered on the power meter median value (bolded) to facilitate visual comparison. Both detectors show excellent agreement at ellipticity values above approximately 0.074, with maximum differences below 0.003. Below this threshold, the photodiode systematically underestimates ellipticity compared to the power meter, likely due to the power meter’s dynamic range adjustment capability which enables more accurate detection at lower signal levels at the cost of measurement speed. The ellipticity range encountered in this work (0.06–0.25) lies predominantly above the 0.074 threshold, ensuring reliable measurements with the photodiode detector.
(TIF)

S5 Fig Linear polarization can become elliptical after metallic mirror reflection. (A) Schematic showing a light ray reflecting from a mirror surface at 45° incidence. The beam’s linear polarization can be decomposed into *s*-polarized (orange arrows, perpendicular to the plane of incidence) and *p*-polarized (blue arrows, parallel to the plane of incidence) components. These components experience different phase shifts upon reflection, potentially introducing ellipticity in the polarization. (B) Phase shift versus angle of incidence for *s* and *p* polarization components reflecting from pure silver at 880 nm (optical constants from https://refractiveindex.info, Shelf:MAIN, Book:Ag (Silver), Page:Jiang et al. 2016). At 45° incidence (highlighted), the phase difference is 13.7°. This phase difference is what causes linear polarization to become elliptical. Calculation code available at https://github.com/Omnistic/fresnel_equations_with_metals. Actual optical simulations (Fig 2 and 4) use protected silver coatings from OpticStudio 2026 R1 (Ansys Zemax).
(TIF)

S6 Fig Temporal stability of the input polarization state. Ellipticity of the polarization state entering the microscope (laser and waveplates, measured at the entrance port before any microscope optics), recorded at a fixed quarter-waveplate and half-waveplate configuration once per minute for two hours. The configuration was chosen to produce an ellipticity near 0.5, within the range where the photodiode-based analyzer measures accurately (well above the 0.074 reliability threshold; see S4 Fig). Over the full two-hour period, the ellipticity remained stable, with a mean of 0.4943 and a standard deviation of 0.0010, confirming that the polarization state delivered to the microscope is stable well beyond the approximately 30 min required for a single two-dimensional ellipticity map acquisition.
(TIF)

S7 FigConvergence of the polychromatic simulation with the number of sampled wavelengths.(A) Simulated ellipticity map (polychromatic, real waveplate retardances, dichroic retardance spanning −20° to +20° around 12.1°) using $N_{\lambda }=31$ wavelengths, taken as the reference case. Unlike Fig 4, only the QWP-near-90° solution obtained by taking the minimum ellipticity from the map at each HWP angle is shown (dashed blue). The solution obtained by fitting the model of Romijn et al. is omitted here for simplicity. (B) Ellipticity along this minimum-ellipticity path, as a function of relative polarization angle, for $N_{\lambda }=1$, 3, 7, 15, and 31 wavelengths (distinguished by color and line style), obtained by subsampling the same underlying set of 31 simulated wavelengths. $N_{\lambda }=15$, used throughout the main text, is shown in bold. (C) Root-mean-square error (RMSE) of the ellipticity map, relative to the $N_{\lambda }=31$ reference, as a function of the number of wavelengths sampled; marker color matches the corresponding curve in panel B. $N_{\lambda }=15$ closely approaches the $N_{\lambda }=31$ reference, supporting its use as a computationally efficient approximation of the full polychromatic simulation throughout this work.(TIF)

S8 FigSensitivity of residual ellipticity to the span of dichroic retardance across the illumination bandwidth.Ellipticity along the minimum-ellipticity compensation path (QWP-near-90° solution), as a function of relative polarization angle, for polychromatic simulations with real waveplate retardances ($N_{\lambda }=15$, 12.5 nm bandwidth), while varying the total span of the dichroic retardance around the central value of 12.1°, from ±0 (i.e., the same retardance at every wavelength) up to ±60° in steps of 10°. Increasing the span of dichroic retardance progressively increases the residual ellipticity along the compensation path. The ellipticity amplitude only approaches the experimental value of approximately 0.24 at the largest span tested (±60°).(TIF)

S9 FigSensitivity of residual ellipticity to the laser spectral bandwidth.Ellipticity along the minimum-ellipticity compensation path (QWP-near-90° solution), as a function of relative polarization angle, for polychromatic simulations with real waveplate retardances ($N_{\lambda }=15$), for four illumination bandwidths: monochromatic, and 2 nm, 12.5 nm, and 25 nm FWHM. The dichroic retardance-versus-wavelength slope was held fixed across all bandwidths, calibrated so that the 12.5 nm case spans ±30° around the central value of 12.1°, which is consistent with the reference condition used elsewhere in this work. Narrower and broader bandwidths therefore sample a correspondingly smaller or larger portion of this same retardance slope. Residual ellipticity increases with bandwidth, which is consistent with narrower-bandwidth sources reducing the polychromatic contribution to residual ellipticity.(TIF)

S1 File SolidWorks assembly files for polarization analyzer. (PDF)

S2 File Python code for data acquisition and analysis. (PDF)

S3 File OpticStudio simulation files and Python automation code. (PDF)

S4 File Experimental ellipticity measurement data. (PDF)

S1 Appendix Angular displacement during three-sample averaging. (PDF)

S2 Appendix Photodiode bandwidth calculation. (PDF)

S3 Appendix ZPL macro implementation for Jones matrix surfaces in OpticStudio. (PDF)

S4 AppendixAnalytical identifiability of $\theta_{0}$ and $\alpha_{0}$ at δ=0.(PDF)

S5 Appendix Ellipticity extraction from OpticStudio simulations. (PDF)

S1 Table System fitting accuracy under ideal and real waveplate retardance. (PDF)
