# Supplementary material for: Persistent residual ellipticity in waveplate-compensated polarization-resolved SHG microscopy on a commercial multiphoton microscope
Source: PLoS One. 2026 Nov 13;21(11):e0357256. doi: 10.1371/journal.pone.0357256 (PMC13766257; doi:10.1371/journal.pone.0357256)
Supplement: Supplementary file 1 [file pone.0357256.s010.pdf]

S1 File. SolidWorks assembly files for polarization analyzer. Archive containing the polarization analyzer CAD assembly shown in S2 Fig. The main files are `polarization_analyzer.sldasm` (assembly) and `polarization_analyzer.slddrw` (drawing). All component models were obtained from the Thorlabs website and are included via SolidWorks Pack and Go. Files were created with SolidWorks Premium 2022 SP5.0 (Dassault Systèmes). Available at `https://doi.org/10.5281/zenodo.17736144`.
