# Supplementary material for: Persistent residual ellipticity in waveplate-compensated polarization-resolved SHG microscopy on a commercial multiphoton microscope
Source: PLoS One. 2026 Nov 13;21(11):e0357256. doi: 10.1371/journal.pone.0357256 (PMC13766257; doi:10.1371/journal.pone.0357256)
Supplement: Supplementary file 2 [file pone.0357256.s011.pdf]

S2 File. Python code for data acquisition and analysis. GitHub repository containing the complete experimental code base for polarization analyzer control, data acquisition, and ellipticity analysis. Includes implementations of fitting routines (Eq 7, Eq 1-3 from Romijn et al. [11]) with numerical stability improvements, polarization parameter calculations, and figure generation scripts. Available at `https://github.com/Omnistic/residual_ellipticity_in_pshg_hardware_and_analysis` and archived at `https://doi.org/10.5281/zenodo.21887161`.
