# Supplementary material for: Persistent residual ellipticity in waveplate-compensated polarization-resolved SHG microscopy on a commercial multiphoton microscope
Source: PLoS One. 2026 Nov 13;21(11):e0357256. doi: 10.1371/journal.pone.0357256 (PMC13766257; doi:10.1371/journal.pone.0357256)
Supplement: Supplementary file 3 [file pone.0357256.s012.pdf]

S3 File. OpticStudio simulation files and Python automation code. GitHub repository containing OpticStudio 2026 R1 (Ansys Zemax) simulation files and Python code for programmatic control and analysis. Includes: (1) ZAR archive with complete OpticStudio lens files implementing Jones matrix simulations for monochromatic and polychromatic cases, including all ZPL macros for Jones matrix surface calculations, (2) Python scripts using ZOS-API and ZOSPy to automate component rotation and data extraction from OpticStudio, and (3) data processing and figure generation scripts for ellipticity analysis. The logic and implementation details of the ZPL macros used in OpticStudio are documented in S3 Appendix and S5 Appendix. Available at `https://github.com/Omnistic/residual_ellipticity_in_pshg_simulations` and archived at `https://doi.org/10.5281/zenodo.21887218`.
