# Supplementary material for: Persistent residual ellipticity in waveplate-compensated polarization-resolved SHG microscopy on a commercial multiphoton microscope
Source: PLoS One. 2026 Nov 13;21(11):e0357256. doi: 10.1371/journal.pone.0357256 (PMC13766257; doi:10.1371/journal.pone.0357256)
Supplement: Supplementary file 4 [file pone.0357256.s013.pdf]

S4 File. Experimental ellipticity measurement data. Complete dataset of experimental measurements including two-dimensional ellipticity maps, baseline measurements, and validation data. Each measurement is stored as a NumPy compressed archive (.npz) with the following structure: `analog_data` contains raw analog input data (2 channels × 9000 samples) from the photodiode signal (channel 0) and motor trigger signal (channel 1) without offset subtraction; `measurement_data` contains processed photodiode intensity as a function of polarization analyzer angle (2 × 360 array); `calibration_mean` contains the dark current offset value used for background subtraction during post-processing; `calibration_std` contains the dark current standard deviation recorded as an indicator of measurement stability during DAQ warm-up. File naming conventions: timestamps with optional descriptions (YYYYMMDDTHHMMSSZ_description.npz format) for individual measurements, HWP and QWP angles (HWP-XXX_QWP-YYY.npz format) for full ellipticity maps, or HWP angle only (XXX.npz format) for HWP-only baseline measurements. Data can be processed using the analysis code in S2 File. Total size approximately 120 MB, available at `https://doi.org/10.5281/zenodo.18433940`.
