# Supplementary material for: Persistent residual ellipticity in waveplate-compensated polarization-resolved SHG microscopy on a commercial multiphoton microscope
Source: PLoS One. 2026 Nov 13;21(11):e0357256. doi: 10.1371/journal.pone.0357256 (PMC13766257; doi:10.1371/journal.pone.0357256)
Supplement: Supplementary file 5 [file pone.0357256.s014.pdf]

S1 Appendix. Angular displacement during three-sample averaging. This appendix calculates the angular displacement of the polarization analyzer rotation stage during the three-sample averaging window used in post-processing.

The data acquisition device samples at 25 000 samples/s, corresponding to a sampling interval of

$$\Delta t = \frac{1}{25\,000\,\mathrm{s}^{-1}} = 40\,\mu\mathrm{s}. \tag{S1.1}$$

Post-processing averages each trigger position sample with its two subsequent samples, spanning a time window of

$$\Delta t_{\mathrm{avg}} = 3 \times 40\,\mu\mathrm{s} = 120\,\mu\mathrm{s}. \tag{S1.2}$$

The rotation stage operates at a maximum speed of 1800 °/s. During the averaging window, the angular displacement corresponds to

$$\Delta\theta = 1800\,{}^{\circ}\,\mathrm{s}^{-1} \times 120\,\mu\mathrm{s} = 0.216^{\circ}. \tag{S1.3}$$

This angular displacement is small compared to the 1° trigger spacing and does not compromise measurement accuracy. The averaging improves signal-to-noise ratio while maintaining adequate angular resolution for ellipticity determination.
