# Supplementary material for: Persistent residual ellipticity in waveplate-compensated polarization-resolved SHG microscopy on a commercial multiphoton microscope
Source: PLoS One. 2026 Nov 13;21(11):e0357256. doi: 10.1371/journal.pone.0357256 (PMC13766257; doi:10.1371/journal.pone.0357256)
Supplement: Supplementary file 6 [file pone.0357256.s015.pdf]

S2 Appendix. Photodiode bandwidth calculation. This appendix provides the calculation for the photodiode bandwidth to verify adequate frequency response for the polarization analyzer measurements.

The bandwidth of a photodiode detector is limited by the junction capacitance C_j and load resistance R_{load} according to:

$$f_{\text{BW}} = \frac{1}{2\pi R_{\text{load}} C_j}. \tag{S2.1}$$

For the SM1PD1A photodiode operating at 10 V bias voltage, the manufacturer (Thorlabs) specifies a typical junction capacitance of $C_j = 328\,\text{pF}$. With the load resistance of $R_{\text{load}} = 500\,\Omega$ used in this work, the bandwidth is calculated as

$$f_{\text{BW}} = \frac{1}{2\pi \times 500\,\Omega \times 328\,\text{pF}} \approx 970\,\text{kHz}. \tag{S2.2}$$

The data acquisition device samples at 25 000 samples/s, corresponding to a Nyquist frequency of 12.5 kHz. The photodiode bandwidth ($f_{\text{BW}} = 970\,\text{kHz}$) well exceeds this requirement. At higher load resistances, such as $R_{\text{load}} = 5\,\text{k}\Omega$, the bandwidth would be approximately $f_{\text{BW}} = 97\,\text{kHz}$, which still satisfies the Shannon-Nyquist criterion with comfortable margin. We recommend maintaining load resistance below $5\,\text{k}\Omega$ to ensure adequate bandwidth, as real-world signal integrity benefits from bandwidths substantially higher than the theoretical Shannon-Nyquist minimum.
