# Supplementary material for: Persistent residual ellipticity in waveplate-compensated polarization-resolved SHG microscopy on a commercial multiphoton microscope
Source: PLoS One. 2026 Nov 13;21(11):e0357256. doi: 10.1371/journal.pone.0357256 (PMC13766257; doi:10.1371/journal.pone.0357256)
Supplement: Supplementary file 7 [file pone.0357256.s016.pdf]

S3 Appendix. ZPL macro implementation for Jones matrix surfaces in OpticStudio. This appendix details the implementation of Jones matrix surfaces in OpticStudio (Ansys Zemax) for modeling linear retarders such as waveplates and dichroic mirrors.

Parameter reduction using physical constraints. OpticStudio's Jones matrix surface type accepts eight parameters: the real and imaginary components of four complex coefficients (A, B, C, D). However, the physical form of a linear retarder given by Eq 1 shows that the matrix can be fully specified by only three independent values, as given in Eq 2. We implemented three ZPL macro solves to automatically calculate these coefficients from the physically meaningful parameters: retardance (Γ) and fast axis angle (β). These macros are embedded in the OpticStudio files and are automatically available when the ZAR archive (S3 File) is opened.

ZPL macro solve implementation. The input parameters for each Jones matrix surface are specified using a comment-based system with dummy surfaces. Each Jones matrix surface is assigned a comment identifier (e.g., "QWP" for quarter-waveplate). Two dummy surfaces are placed immediately before the Jones matrix surface with comments "[IDENTIFIER]_RETARDANCE" and "[IDENTIFIER]_ANGLE" (e.g., "QWP_RETARDANCE" and "QWP_ANGLE"). The ZPL macro searches for these surfaces by parsing comments and reads the thickness values as the retardance (in degrees) and fast axis angle (in degrees), respectively. Since simulations trace a single ray coaxial with the optical axis, the additional optical path length from dummy surface thickness does not affect results, and these surfaces can be hidden in OpticStudio's 3D Layout and Shaded Model views.

Usage and benefits. This implementation enables direct manipulation of retardance and orientation in the OpticStudio interface without requiring ZOS-API or programmatic control. Users can rotate components or adjust retardance by simply modifying the thickness values of dummy surfaces, with all matrix elements updating automatically through the ZPL macro solves. This approach was used to implement the QWP, HWP, and dichroic mirror in all simulations presented in the main text. Complete ZPL macro code and example OpticStudio files are provided in S3 File.

ZPL macro code. The three ZPL macros implement the coefficient calculations from Eq 2. Each macro follows the same structure: parse the comment to identify the surface, locate the dummy surfaces for retardance and angle, and compute the appropriate coefficient.

- Macro for $A_r = \cos(\Gamma/2)$:

```
CONS_PI = 2 * ACOS(0)
TO_RAD = CONS_PI / 180
current_surface_number = SOSO(0)
plate_name$ = $COMMENT(current_surface_number)
# Case-Insensitive
retardance_comment$ = plate_name$ + "_retardance"
retardance = THIC( SURC(retardance_comment$) )
retardance = retardance * TO_RAD
SOLVE
RETURN COSI( retardance/2 )
```

- Macro for $A_i = \sin(\Gamma/2)\cos(2\beta)$:

```
CONS_PI = 2 * ACOS(0)
TO_RAD = CONS_PI / 180
current_surface_number = SOSO(0)
plate_name$ = $COMMENT(current_surface_number)
# Case-Insensitive
retardance_comment$ = plate_name$ + "_retardance"
angle_comment$ = plate_name$ + "_angle"
retardance = THIC( SURC(retardance_comment$) )
angle = THIC( SURC(angle_comment$) )
```

```
retardance = retardance * TO_RAD
angle = angle * TO_RAD
SOLVE
RETURN SINE( retardance/2 ) * COSI( 2*angle )
```

- Macro for $B_i = \sin(\Gamma/2)\sin(2\beta)$:

```
CONS_PI = 2 * ACOS(0)
TO_RAD = CONS_PI / 180
current_surface_number = SOSO(0)
plate_name$ = $COMMENT(current_surface_number)
# Case-Insensitive
retardance_comment$ = plate_name$ + "_retardance"
angle_comment$ = plate_name$ + "_angle"
retardance = THIC( SURC(retardance_comment$) )
angle = THIC( SURC(angle_comment$) )
retardance = retardance * TO_RAD
angle = angle * TO_RAD
SOLVE
RETURN SINE( retardance/2 ) * SINE( 2*angle )
```
