# Supplementary material for: Persistent residual ellipticity in waveplate-compensated polarization-resolved SHG microscopy on a commercial multiphoton microscope
Source: PLoS One. 2026 Nov 13;21(11):e0357256. doi: 10.1371/journal.pone.0357256 (PMC13766257; doi:10.1371/journal.pone.0357256)
Supplement: Supplementary file 8 [file pone.0357256.s017.pdf]

S4 Appendix. Analytical identifiability of θ_0 and α_0 at $\delta = 0$. To investigate the origin of the θ_0 and α_0 discrepancy observed between the entrance port and sample plane (Table 3), we symbolically simplified the intensity model of Romijn et al. (Eq 1-3) in the limiting case $\delta = 0$, using Mathematica 13.3 (Wolfram Research). Since I_0 is a pure multiplicative prefactor, it was set to 1 without loss of generality. The value of γ was set to 1, which is consistent with the small diattenuation expected for protected silver mirrors near 45° incidence in the near-infrared, and with the values obtained from fitting our experimental data (0.99 to 1.07 across all measurement locations and experiments; Table 3). Under these conditions, the model simplifies to:

$$I(\theta, \phi, \alpha)\Big|_{\delta=0,\ \gamma=I_0=1} = \frac{1}{4}\left[\cos\Big(2\big(\alpha - 2\theta\big) + 4\phi\Big) + \cos\Big(2\big(\alpha - 2\theta\big)\Big) + 2\right]. \tag{S4.1}$$

Both cosine arguments in Eq S4.1 depend on θ and α only through the combination $\alpha - 2\theta$, never separately. Since $\theta = \theta' - \theta_0$ and $\alpha = \alpha' - \alpha_0$, where θ' and α' are the motor angles set during a measurement, this means that for any sequence of measurements performed at fixed motor angles, the predicted intensity is identical whether the true offsets are (θ_0, α_0) or $(\theta_0 + t, \alpha_0 + 2t)$, for any t. Consequently, θ_0 and α_0 cannot be individually identified at $\delta = 0$, only their combination $\alpha_0 - 2\theta_0$ can be recovered from fitting. In contrast, ϕ appears independently, so ϕ_0 remains separately identifiable.

The Mathematica code used to derive Eq S4.1 is shown below.

```
IntensityModel[theta_, phi_, alpha_, delta_, gamma_, I0_] :=
 Module[{u, d1, d2, d3, d4},
  u = 2 theta - phi;
  d1 = -gamma (Cos[delta] Sin[phi] Sin[u]
      + Sin[delta] Cos[phi] Cos[u]);
  d2 = -gamma (Sin[delta] Sin[phi] Sin[u]
      - Cos[delta] Cos[phi] Cos[u]);
  d3 = Sin[phi] Cos[u];
  d4 = Cos[phi] Sin[u];
  I0 ((d1^2 + d2^2) Cos[alpha]^2
      + (d3^2 + d4^2) Sin[alpha]^2
      + 2 (d1 d3 + d2 d4) Sin[alpha] Cos[alpha])
 ]

result = FullSimplify[
   IntensityModel[theta, phi, alpha, 0, 1, 1]
 ];

Print[TraditionalForm[result]]
```
