# Supplementary material for: Persistent residual ellipticity in waveplate-compensated polarization-resolved SHG microscopy on a commercial multiphoton microscope
Source: PLoS One. 2026 Nov 13;21(11):e0357256. doi: 10.1371/journal.pone.0357256 (PMC13766257; doi:10.1371/journal.pone.0357256)
Supplement: Supplementary file 9 [file pone.0357256.s018.pdf]

S5 Appendix. Ellipticity extraction from OpticStudio simulations. This appendix describes how ellipticity and polarization angle are extracted from OpticStudio simulations for monochromatic and polychromatic cases.

Monochromatic simulations. An ideal linear polarizer, oriented at angle α and rotated over 360°, is added at the end of the optical path to replicate the polarization analyzer used experimentally. The polarizer angle sampling was chosen according to the complexity of each simulation: for the single-parameter HWP sweep (Fig 2B), the polarizer was sampled every 1°, while for the full HWP-QWP-wavelength parameter sweeps (Fig 4 and associated sensitivity analyses), it was sampled every 8° to keep the overall simulation computationally tractable. This coarser sampling (45 points over the full rotation) remains substantially finer than that used in the original experimental implementation of Romijn et al. (6 points over 150°; see Materials and methods), which was already sufficient to recover the fitted parameters reliably. The polarizer is implemented as a Jones matrix surface with the form:

$$J(\alpha) = \begin{pmatrix} A_r & B_r \\ B_r & 1 - A_r \end{pmatrix}, \tag{S5.1}$$

where

$$\begin{aligned} A_r &= \cos^2 \alpha, \\ B_r &= \cos \alpha \sin \alpha. \end{aligned} \tag{S5.2}$$

A_r and B_r are implemented as independent ZPL macro solves, and the remaining matrix elements are implemented as pickups. As in S3 Appendix, a dummy surface is placed immediately before the Jones matrix surface, with its thickness interpreted as the polarizer angle (in degrees); this makes the polarizer angle directly readable and adjustable from the OpticStudio interface.

- Macro for $A_r = \cos^2 \alpha$:

```
CONS_PI = 2 * ACOS(0)
TO_RAD = CONS_PI / 180
angle_deg = THIC( SURC("linear_polarizer_angle") )
angle_rad = angle_deg * TO_RAD
SOLVE
RETURN COSI(angle_rad) * COSI(angle_rad)
```

- Macro for $B_r = \cos \alpha \sin \alpha$:

```
CONS_PI = 2 * ACOS(0)
TO_RAD = CONS_PI / 180
angle_deg = THIC( SURC("linear_polarizer_angle") )
angle_rad = angle_deg * TO_RAD
SOLVE
RETURN COSI(angle_rad) * SINE(angle_rad)
```

OpticStudio records the transmitted intensity as a function of polarizer angle using the CODA Merit function operand with Data = 0 ("relative transmitted polarized intensity", default parameters). The ellipticity and polarization angle are then computed from this simulated intensity curve by fitting Eq 7 from Romijn et al. [11], using the same fitting procedure as for experimental data (see Materials and methods). The corresponding code is provided in S3 File.

Polychromatic simulations. This same rotating-polarizer approach is applied independently at each wavelength using a multi-configuration setup in OpticStudio, with one configuration per wavelength, and the transmitted intensity is extracted at each polarizer angle for every configuration. The per-wavelength weights are calculated in the Python simulation code and used to sum the intensity curves incoherently across wavelengths, simulating the detector response to broadband illumination, before ellipticity and polarization angle are extracted as described above.
