# Supplementary material for: Persistent residual ellipticity in waveplate-compensated polarization-resolved SHG microscopy on a commercial multiphoton microscope
Source: PLoS One. 2026 Nov 13;21(11):e0357256. doi: 10.1371/journal.pone.0357256 (PMC13766257; doi:10.1371/journal.pone.0357256)
Supplement: Supplementary file 10 [file pone.0357256.s019.pdf]

S1 Table. System fitting accuracy under ideal and real waveplate retardance. Errors in the fitted parameters ($\Delta\theta_0$, $\Delta\phi_0$, $\Delta\alpha_0$) relative to their ground-truth values (18°, 11°, and 33°, respectively), obtained at 880 nm from simulated ellipticity maps generated using ideal waveplate retardances (0.25 λ for the QWP, 0.5 λ for the HWP) compared to manufacturer-specified real retardances (0.258 λ and 0.516 λ, respectively). Maps were computed with the HWP and QWP each sampled at 2° intervals, and the polarizer sampled at 6° intervals (60 steps over 360°). This sampling is coarser than what was used elsewhere in this work (see S5 Appendix), and was chosen to keep the repeated simulation runs described below computationally tractable. For each waveplate condition, the dichroic mirror retardance was chosen to produce a system retardance δ comparable to either the entrance port or the sample plane value reported in the experimental measurements (Fig 3A). Each value is the mean of 5 simulation runs. The standard deviation across these 5 runs was exactly 0 for every entry in the table and is therefore omitted from the cells for readability. Introducing real waveplate retardance substantially degrades the accuracy of the recovered θ_0 and α_0 parameters, while ϕ_0 remains well fitted regardless. The larger error observed at the lower system retardance (entrance port) compared to the higher one (sample plane) is consistent with the analytically confirmed degeneracy of θ_0 and α_0 at $\delta = 0$ (S4 Appendix). We have however not characterized how the error scales with δ away from this limiting case. †System retardance arising from the five silver-protected mirrors alone, without any dichroic mirror or waveplates. In the ideal waveplate case, the system retardance is exactly the sum of this mirrors-only retardance and the ground-truth dichroic retardance.

		**DC Ret (°)**	**δ (°, mean)**	**$\Delta\theta_0$ (°, mean)**	**$\Delta\phi_0$ (°, mean)**	**$\Delta\alpha_0$ (°, mean)**
Mirrors only†		0	−32.6468	0	0	0
Ideal	Entrance	28	−4.6468	0	0	0
	Sample	18	−14.6468	0	0	0
Real	Entrance	28	−3.8399	−12.1164	−0.0008	−24.2805
	Sample	18	−12.8350	−3.2056	0.0053	−6.5994
