# Supplementary material for: Persistent residual ellipticity in waveplate-compensated polarization-resolved SHG microscopy on a commercial multiphoton microscope
Source: PLoS One. 2026 Nov 13;21(11):e0357256. doi: 10.1371/journal.pone.0357256 (PMC13766257; doi:10.1371/journal.pone.0357256)
Supplement: Supplementary file 11 [file pone.0357256.s021.pdf]

This manuscript investigates the issue of residual ellipticity in waveplate-compensated polarization-resolved second harmonic generation microscopy, proposing that the spectral bandwidth of the femtosecond laser is the primary limiting factor. Upon review, the manuscript exhibits five major flaws concerning experimental validation, theoretical simulation, and model rigor.

First, the core hypothesis lacks direct experimental data support. The authors claim that residual ellipticity originates from the combination of the wavelength-dependent retardance of the dichroic mirror and the broadband spectrum of the laser. However, they explicitly admit to not measuring the actual retardance spectrum of the Leica microscope dichroic mirror used, instead directly inferring it from referenced data. This unverified substitution weakens the direct persuasiveness of the conclusions.

Second, there is a significant quantitative discrepancy between the Jones matrix simulation results and the experimental data. The residual ellipticity amplitude derived from the polychromatic light simulation is only around 0.13, whereas the actual experimental measurement is as high as 0.25. Although the authors mention this difference, they fail to further refine the physical model to bridge this gap, indicating that the current theoretical explanation is quantitatively incomplete.

Third, the parameter settings for the theoretical simulations are disconnected from the actual experimental equipment. The Mai Tai femtosecond laser used in the experiment has a nominal bandwidth of 12.5 nm, yet the authors employed a bandwidth of 20 nm in the simulation. The practice of artificially relaxing parameters to highlight spectral effects lacks rigor. Furthermore, even with the expanded bandwidth parameter, the simulated polarization distortion remains far below the true experimental value.

Fourth, the established optical system model is overly simplified and ignores key optical components. The simulation only includes waveplates, five metal mirrors, and a dichroic mirror, completely omitting the high numerical aperture objective lens. High numerical aperture objectives are known to cause severe polarization distortions. Excluding the most critical imaging component of the microscope results in a simulation environment that fails to reflect the complexity of the optical path at the sample plane.

Fifth, there are issues with the applicability of the fitting model, resulting in fitting parameters that lack a reasonable physical basis. According to the supplementary data, the initial angle parameter of the half-wave plate exhibits a massive shift from 18.07 degrees to 25.61 degrees between the entrance port and the sample plane. Since the physical mounting position of the half-wave plate remained unchanged, this parameter jump is illogical. The authors simply attribute this to the model's inability to fully capture the sources of residual ellipticity, exposing a lack of robustness in their mathematical fitting model when handling real system errors.
