# Supplementary material for: Persistent residual ellipticity in waveplate-compensated polarization-resolved SHG microscopy on a commercial multiphoton microscope
Source: PLoS One. 2026 Nov 13;21(11):e0357256. doi: 10.1371/journal.pone.0357256 (PMC13766257; doi:10.1371/journal.pone.0357256)
Supplement: Supplementary file 12 [file pone.0357256.s022.pdf]

# Reviewer 1

> *This is a meticulously conducted and highly transparent study that addresses a critical challenge in polarization-resolved second harmonic generation microscopy. The authors honestly report their experimental difficulties in achieving perfect linear polarization control, despite implementing a state-of-the-art waveplate compensation method on a commercial microscope. Their systematic documentation of hardware modifications, careful measurement of residual ellipticity (up to 0.25), and insightful simulation linking the problem to wavelength-dependent birefringence and broad femtosecond bandwidth are particularly commendable. The conclusion that conventional compensation may be fundamentally limited for broadband pulses is an important cautionary insight for the field. Moreover, their decision to share open-source analysis code and simulation files sets an excellent example of reproducible and collaborative science. This work will undoubtedly guide future researchers toward alternative strategies, such as narrow-bandwidth picosecond sources or specimen rotation, and prevents many from wasting effort on unattainable compensation.*

We thank Reviewer 1 for their careful reading and positive assessment of our work. We are grateful that the reviewer found value in the reporting of the experimental difficulties we encountered, and in our systematic documentation of the hardware implementation and residual ellipticity measurements. We also appreciate the recognition of our commitment to transparency and reproducibility.

In response to comments from the other reviewers, we have moderated the framing of the proposed spectral-bandwidth mechanism throughout the manuscript and revised the title accordingly. We trust these changes do not affect the strengths of the manuscript that Reviewer 1 highlighted.

We hope that our work proves useful to the community, and we thank the reviewer for their time and consideration.

# Reviewer 2

## Major Comment 1

> *The Central Mechanistic Claim Lacks Direct Experimental Validation*
>
> *The paper's core hypothesis — that residual ellipticity arises from wavelength-dependent dichroic mirror birefringence combined with femtosecond pulse bandwidth — is supported only by simulation, not by direct measurement. The authors acknowledge that direct measurement of the dichroic mirror retardance*

*spectrum 'would be required for quantitative validation but is beyond the scope of this work.' This is a significant limitation for a paper whose title asserts this mechanism as the explanation. The manuscript never actually measures the spectral retardance of the specific dichroic mirror in the Leica SP8 system; instead it borrows values from Schon et al. (2008), which were obtained on a different system. Without at least a qualitative experimental test — for example, comparing ellipticity residuals when using a narrowband (picosecond) versus broadband (femtosecond) source at the same center wavelength — the causal claim remains speculative. The authors should either perform this comparison, characterize their dichroic mirror directly using ellipsometry or the Mueller matrix method, or substantially moderate the language of their title and conclusions to reflect that the mechanism is proposed rather than demonstrated.*

We thank the reviewer for this important comment. Our proposed mechanism is indeed supported by simulation rather than direct measurement, and we fully agree that a direct characterization of the dichroic mirror (e.g., by ellipsometry or Mueller matrix methods) or a comparison between broadband femtosecond and narrowband picosecond sources at the same center wavelength would provide the definitive test of our hypothesis. Unfortunately, neither is feasible within the present study: the dichroic mirror is continuously used on a shared facility instrument and cannot be removed for ellipsometry, and we do not have access to a picosecond source at the relevant wavelength. We have therefore framed these measurements explicitly as the necessary next steps for validating the proposed mechanism, rather than as work falling outside the scope of the paper.

Consequently, we have adopted the third option suggested by the reviewer, namely, to substantially moderate the language throughout the manuscript so that the spectral-bandwidth mechanism is presented as a proposed explanation rather than a demonstrated one.

We would also like to clarify the central aim of our study, as we realize it may not have been stated sufficiently clearly in the original submission. The primary result of this work is a reproducibility finding: we implemented a published state-of-the-art compensation method on the same type of commercial instrument used in the original study and observed that it provided essentially no practical improvement in our system. We believe that this empirical observation stands as a valuable result, independently of the mechanism we propose to explain it. The mechanistic hypothesis is offered as a plausible direction for future investigation, not as a validated conclusion.

Accordingly, we have made the following changes:

- The title has been revised from *"Residual ellipticity in waveplate-compensated polarization-resolved SHG microscopy may arise from femtosecond laser*

*spectral bandwidth"* to *"Persistent residual ellipticity in waveplate-compensated polarization-resolved SHG microscopy on a commercial multiphoton microscope,"* removing the mechanistic claim from the title and only presenting the spectral-bandwidth hypothesis as a proposed explanation in the abstract and discussion.

- Throughout the abstract, results, and conclusions, we have replaced assertive phrasing with language reflecting that the mechanism is proposed but not verified (e.g., "may arise from," "we hypothesize," "offers support for the hypothesis," "a possible, though not fully verified, explanation").
- We have added a statement in the discussion making explicit that direct measurement of our dichroic mirror's spectral retardance would be required to confirm the mechanism we propose, and that this remains an open question.

## Major Comment 2

> *Quantitative Mismatch Between Simulation and Experiment Is Insufficiently Addressed*
>
> *The polychromatic simulation produces residual ellipticity of approximately 0.13, while the experimental observations reach up to 0.25 — a factor of nearly two. While the authors list several possible contributing factors (simplified linear retardance model, uncertainty in the dichroic spectrum, other wavelength-dependent elements), no quantitative sensitivity analysis is performed to determine which factor is dominant or whether the proposed mechanism is even sufficient to account for the observed magnitude. This discrepancy is non-trivial: it opens the possibility that other unaccounted mechanisms — e.g., objective lens polarization aberrations, scan mirror retardance, polarization-dependent transmission in optical coatings — may contribute comparably or even dominantly. A parametric analysis showing how the simulated ellipticity amplitude changes with dichroic retardance slope, bandwidth, or the addition of further optical elements would substantially strengthen the paper's argument.*

We thank the reviewer for this comment. To assess whether the proposed mechanism can plausibly account for the observed magnitude of residual ellipticity, and whether other elements might contribute comparably, we have now added a parametric sensitivity analysis to the manuscript (new Supplementary Figures S8 and S9).

Specifically, we varied (1) the total span of dichroic retardance across the illumination bandwidth, from 0° (i.e., the same retardance at every wavelength) up to ±60° in steps of 10° (Supplementary Figure S8), and (2) the illumination bandwidth itself (monochromatic, 2 nm, 12.5 nm, and 25 nm FWHM), at a fixed retardance-versus-wavelength slope (Supplementary Figure S9). The simulated ellipticity amplitude only exceeded 0.2, reaching approximately 0.22, at the most extreme span tested (±60°, i.e.

120° total) over our laser's 12.5 nm bandwidth — approaching but not fully reproducing the experimental maximum of approximately 0.24. More moderate, arguably more plausible spans produced substantially smaller residual ellipticity. Consequently, we have revised the discussion to state the limitation of our simulations explicitly and emphasize the qualitative nature of their agreement with our experimental observations.

We also note that, in preparing this revision, we adopted the more precise experimental maximum of 0.24 (rather than the rounded value of 0.25 used in the original submission) when directly comparing simulated and experimental amplitudes, since this comparison benefits from the added precision. The rounded value of "up to 0.25" is retained elsewhere in the manuscript where it refers more generally to the overall range of ellipticity values observed across all measurements (e.g., the HWP-only baseline).

Regarding whether other optical elements might contribute comparably or dominantly, as detailed in our response to Major Comment 4, no objective lens was present in the beam path for any measurement reported in the paper. Therefore, no objective-induced polarization aberrations can contribute to the residual ellipticity we report. Regarding scan mirrors and other metallic reflective elements, the 0° span condition in our new Supplementary Figure S8 (dichroic retardance held constant across wavelength) isolates any residual polychromatic contribution from the mirror cascade alone since it removes the dichroic's wavelength dependence while leaving the weak, physically expected wavelength dependence of the metallic mirrors' own retardance. This residual is negligible (<0.025) and difficult to discern in the corresponding curve, indicating that mirrors of the kind modeled here are unlikely to account for the observed magnitude on their own. We cannot, however, exclude contributions from other internal optical elements whose construction and coatings are not documented by the manufacturer, and we have added a statement to this effect in the discussion.

## Major Comment 3

> *The Simulation Model Makes Unverified Simplifying Assumptions*
>
> *The polychromatic Jones matrix simulation models the dichroic mirror retardance as varying linearly with wavelength from -12.9 deg to +37.1 deg over 40 nm. This linearity assumption is never justified. The reference measurement from Schon et al. (their Figure 6b) shows that dichroic retardance can exhibit strongly nonlinear, even oscillatory, spectral dependence depending on coating design. The actual spectral retardance of the Leica SP8 dichroic mirror is unknown. Furthermore, the simulation uses nine discrete wavelengths with a Gaussian weighting to represent the femtosecond pulse spectrum, but no justification is given for the adequacy of this sampling. The authors should: (1) test sensitivity of the simulation output to the retardance profile shape (linear vs.*

*nonlinear), (2) verify convergence of the ellipticity estimate with respect to number of spectral samples, and (3) explicitly acknowledge that the borrowed dichroic values may not represent their instrument.*

We thank the reviewer for these three specific points, which we address in turn.

(1) Sensitivity of the simulation output to the retardance profile shape.
We agree that dichroic retardance can, in principle, vary nonlinearly or even non-monotonically with wavelength, as shown in Schön et al.'s Figure 6b for their specific coating. However, because the actual spectral retardance profile of our own dichroic mirror is unknown, any nonlinear profile we might choose to test would be arbitrary and would, in effect, result in fitting noise. We have instead chosen to test sensitivity to the overall span of a linear profile (Supplementary Figure S8) and to the illumination bandwidth (Supplementary Figure S9), which we believe conveys more transparently the uncertainty without introducing additional unverified assumption. To support future work that does have access to a measured retardance profile, we also provide the full simulation code (Supplementary File S3) so that a specific, empirically derived profile could be substituted directly.

(2) Convergence of the ellipticity estimate with respect to the number of spectral samples.
We have added a new supplementary figure (Supplementary Figure S7) directly addressing this point. Using a reference simulation with 31 sampled wavelengths, we computed the root-mean-square error of the resulting ellipticity map for coarser samplings (1, 3, 7, and 15 wavelengths) relative to this reference. This analysis showed that 7 wavelengths already closely approximate the 31-wavelength reference. To be conservative, we nonetheless increased the number of wavelengths used throughout the revised manuscript from 9 (used in the original submission) to 15 and state this convergence justification explicitly at the first mention of the wavelength sampling.

(3) Acknowledging that the borrowed dichroic values may not represent our instrument.
While we had already noted this limitation in the discussion, we have strengthened the text in the revision to state explicitly that our own dichroic mirror is likely to differ substantially from the one measured by Schön et al. and that their data, being of limited spectral resolution and obtained on a different system, cannot be taken as a reliable estimate of our specific mirror's retardance slope.

## Major Comment 4

*The Role of Objective Lens Polarization Distortions Is Dismissed Too Quickly*

*The authors do not specify the objective NA used, nor do they provide any quantification of its contribution to the observed ellipticity. Given that the paper attributes residual ellipticity specifically to the dichroic-bandwidth interaction,*

*the absence of a systematic assessment of the objective lens contribution — particularly since measurements at the entrance port (ellipticity < 0.05) vs. sample plane (up to 0.25) show a significant increase through the microscope body — weakens the attribution. The authors should at minimum report the objective NA, reference appropriate literature on objective-induced polarization effects, and discuss whether this contribution has been ruled out or quantified.*

We thank the reviewer for raising this point, and we apologize that our description was not sufficiently clear on this important detail. We would like to clarify that no objective lens was present in the beam path for any of the measurements reported in the manuscript. As in the calibration procedures of Romijn et al. and Chou et al., the objective lens is removed and the polarization is measured at the sample plane (i.e., the position where the sample would normally be located). The "sample plane" measurements therefore characterize the polarization of the beam delivered to the objective, not the polarization after transmission through an objective lens.

Consequently, objective-induced polarization effects do not contribute to the residual ellipticity we report, and the increase in ellipticity from the entrance port (< 0.05) to the sample plane (up to 0.25) is attributable to the intervening optics within the microscope body (mirrors and the dichroic mirror), not to the objective. We agree entirely that high-NA objectives can introduce significant polarization distortions, and this is precisely why the calibration is performed without the objective in place, thus isolating the contribution of the upstream optics.

We recognize that this was not stated explicitly enough in our original manuscript, and that the term "sample plane" may have implied measurement through the full imaging path including the objective. To prevent this misunderstanding, we have revised the Materials and Methods and the relevant Results Sections text to state clearly that all measurements were performed at the sample plane with the objective removed, consistent with the methodology of Romijn et al. and Chou et al. We have also added a statement to the Conclusions noting that, as a result, the present study characterizes the polarization state delivered to the objective and does not address objective-induced depolarization, which would be a further consideration for full-system polarization accuracy in high-NA imaging.

## Major Comment 5

*Statistical Characterization of Ellipticity Measurements Is Absent*

*The paper presents ellipticity curves and maps as single-instance measurements without any statistical characterization — no error bars, no repeated measurement variability, no confidence intervals. The authors mention that their polarization analyzer takes 360 ms per measurement and that validation was done against a power meter (S4 Fig), but the reproducibility of the*

*two-dimensional ellipticity maps (requiring 30 minutes of acquisition) is never assessed. Questions that remain unanswered include: How stable is the laser polarization state over 30 minutes? Does the ellipticity map change between acquisition runs? What is the measurement noise floor for the 2D maps? The authors should provide repeated measurements of at least the compensation curve (Fig. 3B) with appropriate error characterization, and discuss measurement stability over the full acquisition duration.*

We thank the reviewer for this comment. We agree that the original submission did not adequately characterize measurement reproducibility and stability, and we have added the following analyses to address the three specific questions raised.

<u>1. Reproducibility of the ellipticity maps between acquisition runs.</u>
We repeated the full map acquisition and fitting procedure on two additional, separate days (new Table 3, Exp 2 and 3), at the same angular sampling as Exp 1, including three separate map acquisitions at the sample plane within a single session (Exp 3, Sample 1-3). Across all three experiments, the fitted QWP orientation angle remained remarkably stable (within approximately 0.9° at the sample plane, and 1.0° at the entrance port), while the fitted HWP and polarization analyzer angles showed a consistent discrepancy between the entrance port and sample plane in every experiment, with some additional spread between experiments. The three repeated acquisitions within Exp 3 (Sample 1-3) agree closely with one another (system retardance within 0.2°, HWP and analyzer angles within 0.2°), demonstrating that, for a fixed physical setup, our fitting procedure is highly reproducible.

We note that the fitted system retardance $\delta$ at the sample plane varies only modestly across experiments performed on separate days (approximately 11.0° to 13.9° across Exp 1–3). As the instrument is shared with other users, the waveplates are removed from their mounts and reinstalled between sessions, which plausibly accounts for this small variation. Separately, in this revision we analytically examined the Romijn et al. model in the idealized limit ($\delta \to 0, \gamma = 1$) and confirmed that the HWP and polarization analyzer angles ($\theta_0, \alpha_0$) become weakly identifiable as system retardance decreases (new Supplementary Table S1 and Appendix S4). While this idealized analysis does not by itself quantitatively account for our specific fitted values, it identifies a structural limitation of the model that is consistent with, and offers a plausible contributing explanation for, the entrance-port/sample-plane discrepancy in these angles. We discuss this further in our response to Reviewer 4's Major Comment 5.

<u>2. Measurement noise floor.</u>

The noise floor of our ellipticity measurement as a function of absolute ellipticity was already characterized in the original submission's Supplementary Figure S4, which we did not modify in this revision. It reports repeated measurements (10–20 per condition)

across a range of ellipticity values using both the photodiode detector used throughout this work and, for validation, a power meter. The spread among repeated photodiode measurements alone is small at each condition, and above the reliability threshold of approximately 0.074, agreement between the photodiode and the power meter is within 0.003 — both small compared to the residual ellipticity amplitudes (up to 0.25) that are the subject of this work. We apologize that this was not made sufficiently clear in the original text; we have now added a sentence in the main text explicitly pointing to S4 Fig as the noise-floor characterization for these measurements.

<u>3. Stability of the polarization state over the acquisition duration.</u>
To assess stability over the full acquisition window, we recorded the ellipticity of the polarization state entering the microscope (laser and waveplates, measured at the entrance port) at a fixed waveplate configuration, once per minute for two hours (which is well beyond the approximately 30-minute duration of a single map acquisition). The configuration was set to produce an ellipticity near 0.49, in a range where the analyzer measures accurately (i.e., well above the 0.074 threshold). The measured ellipticity was highly stable over the full two-hour period, with a mean of 0.4943 and a standard deviation of 0.0010 (0.2% relative; Supplementary Figure S6). This confirms that the polarization state delivered to the microscope is stable over the acquisition window, excluding source drift as a contributor to the observed residual ellipticity. Together with the reproducibility of the three back-to-back sample-plane acquisitions in Exp 3 described above, these results argue against systematic drift in the polarization state during map acquisition.

Together, these additions provide repeated measurements across three independent experiments with quantified reproducibility of all fitted parameters, characterize the measurement noise floor, and address polarization stability over the acquisition duration, as requested.

## Major Comment 6

> *The Practical Significance Threshold Is Not Adequately Justified*
>
> *The authors state that 'for some quantitative polarization-resolved measurements, a residual ellipticity of 0.25 may be acceptable depending on the required measurement precision.' This statement risks being misleading without context. No quantitative analysis is provided linking a given ellipticity value to resulting errors in downstream SHG analysis parameters (e.g., orientation angle error, molecular order parameter bias). The paper would be considerably strengthened by including either an analytical or numerical estimate of how ellipticity of 0.06-0.25 propagates into errors in the polarization-dependent SHG intensity ratio or fitted structural parameters. A worked example or sensitivity*

*analysis connecting ellipticity magnitude to SHG measurement error is strongly recommended.*

We thank the reviewer for this comment, which we have addressed by narrowing the scope of our claims rather than by adding a downstream-error analysis. We recognize that the original statement regarding the acceptability of a given ellipticity value was insufficiently supported, and that a quantitative link between residual ellipticity and errors in downstream SHG parameters would require dedicated analysis and validation that fall outside the scope of this study. Accordingly, we have removed the statement that a residual ellipticity of 0.25 "may be acceptable," and we have revised the surrounding text to make explicit that this work characterizes the polarization state itself and does not assess the propagation of residual ellipticity into downstream SHG analysis. We note that our central finding, namely that that waveplate compensation provided minimal practical improvement in our system, stands regardless of any specific application-acceptability threshold.

## Major Comment 7

*Alternative Explanations for the Two-Peak Residual Pattern Are Not Ruled Out*

*The two-peak periodic structure of the residual ellipticity along the compensation curve (Fig. 3B) is attributed to the polychromatic mechanism. However, this same two-peak structure could in principle arise from: (a) imperfect waveplate retardance calibration (the HWP is specified at 0.516 lambda instead of 0.5 lambda), (b) waveplate angular misalignment or wobble during rotation, (c) beam walk-off during HWP rotation, or (d) residual effects from the five silver mirrors not captured by the assumed model. The monochromatic simulation already shows that non-ideal waveplate retardances introduce asymmetry (Fig. 2B). The authors should explicitly demonstrate — through simulation or experimental controls — that these alternative explanations are insufficient to account for the observed residual ellipticity pattern before attributing it to the spectral bandwidth mechanism.*

We thank the reviewer for this thorough enumeration of alternative explanations, which we address in turn.

(a) Imperfect waveplate retardance calibration.
This alternative is directly testable within the proposed simulation framework: with monochromatic illumination and real, manufacturer-specified retardance for both the HWP (0.516λ) and QWP (0.258λ), the compensation curve obtained by fitting the model of Romijn et al. shows a two-peak pattern with an amplitude of only approximately 0.05 (Figure 4C-D), which is substantially smaller than the approximately 0.24 observed experimentally. Moreover, this residual is not an unavoidable consequence of the real waveplate retardances themselves since taking the minimum ellipticity directly from

the same simulated map, rather than the compensation angles predicted by the fitted model, recovers a curve that remains essentially at zero (Figure 4C-D). This indicates that the residual reflects a limitation of the fitting procedure at low system retardance (see also our response to Major Comment 5 and the new Supplementary Table S1 and Appendix S4) rather than a fundamental physical consequence of non-ideal waveplate retardance. Non-ideal waveplate retardance is therefore a real, but clearly insufficient and largely correctable, contributor to the observed residual on its own.

(b) Waveplate angular misalignment or wobble, and (c) beam walk-off during HWP rotation.
We unfortunately do not have a dedicated experimental control allowing us to isolate these mechanical effects in situ and therefore cannot rule out some contribution from them. We note, however, that the manufacturer-specified tolerances of our hardware are small: the HWP and QWP rotation stages (Thorlabs KPRM1E/M) have a specified wobble of 200 µrad (~0.011°), the polarization analyzer rotation stage (Thorlabs DDR25/M) has a specified wobble of 1790 µrad (~0.103°), and the waveplates themselves (Thorlabs AQWP10M-980, AHWP10M-980) have a specified beam deviation of less than 10 arcsec (~0.003°). While we cannot verify that our system performs at these nominal specifications throughout its full range of motion, we have no reason to believe our setup is substantially misaligned or unstable beyond this. These tolerances are small enough that we would not expect them, on their own, to be the dominant source of the residual ellipticity pattern we report. We further note that our simulation contains no mechanical imperfections of this kind at all (waveplate rotation is modeled as ideal), and yet incorporating only the polychromatic nature of the illumination reproduces the qualitative two-peak, two-valley pattern and residual ellipticity of comparable order of magnitude to the experimental observations (Figure 4). We further note that a qualitatively similar two-peak residual pattern is already present in the originally published data of Romijn et al. (their Figure 6b), obtained on different hardware. We consider it unlikely that two independent implementations would coincidentally share the same mechanical misalignment artifacts and therefore take it as indirect evidence for a shared physical origin rather than an implementation-specific mechanical defect.

(d) Residual effects from the mirror cascade not captured by the assumed model.
We have added a direct test of this alternative as part of our response to Major Comment 2: the polychromatic simulation at zero dichroic retardance span (i.e., constant retardance across wavelength, isolating any wavelength-dependence intrinsic to the mirror cascade alone) shows negligible residual ellipticity (new Supplementary Figure S8, span = 0 condition), indicating that the mirror cascade, as modeled, cannot account for the observed residual on its own.

Taken together, we believe that these analyses strengthen the case that the polychromatic dichroic mechanism, rather than the alternatives raised, is the dominant contributor to the observed two-peak residual pattern. We however continue to acknowledge (as per our response to Major Comment 2) that we cannot fully exclude contributions from other, undocumented wavelength-dependent elements in our closed, commercial system.

## Major Comment 8

> *The Proposed Alternative Solutions Are Insufficiently Analyzed*
>
> *The conclusions recommend specimen rotation and picosecond laser sources as alternatives, but neither is analyzed quantitatively in terms of its own practical limitations. Picosecond lasers reduce SHG efficiency (signal scales as peak power squared for pulsed excitation), which could be significant for weakly scattering biological specimens. The authors should provide at least a brief quantitative estimate of the SHG signal penalty expected when switching from femtosecond to picosecond pulses at the same average power, and discuss whether this tradeoff is acceptable for their targeted applications. Similarly, specimen rotation introduces translation-rotation coupling that the authors acknowledge but do not quantify in terms of required mechanical precision.*

We thank the reviewer for this comment. We agree that both alternatives carry practical tradeoffs, which we qualitatively noted in our original Conclusions Section: for picosecond sources, we state that "the longer pulse duration would reduce SHG generation efficiency," and for specimen rotation, we note that it "introduces technical challenges in maintaining the field of view relative to the rotation axis, necessitating motorized x-y translation and rotation stages." These two paragraphs are intended as pointers toward directions worth exploring, rather than as a feasibility study for either approach. A full quantitative analysis of either alternative would depend heavily on the specific instrument, laser source, and application in question, and is beyond the scope of a study whose primary contribution is to characterize the compensation method itself.

Regarding the picosecond laser tradeoff specifically: while SHG, like two-photon excited fluorescence, depends on the square of the instantaneous intensity, and is therefore expected to show a comparable reduction in efficiency at longer pulse durations, we are not aware of a directly applicable, published quantitative treatment for SHG specifically. We are therefore reluctant to present a numerical estimate that we cannot properly support. We believe that stating the qualitative direction and magnitude of this tradeoff, as we already do, more accurately reflects the current state of evidence than an unsupported number would.

Similarly, we have not added a numerical estimate for the mechanical precision required for specimen rotation, since this depends entirely on instrument-specific parameters (eccentricity of the rotation axis, desired field-of-view registration tolerance) that we have not characterized for our system and that would vary substantially between implementations. We believe that stating this dependency explicitly in the text, as we already do, is more informative than an arbitrary number.

## Minor Comments (1-12)

1. *Title precision: The phrase 'may arise' is appropriately cautious, but given that the paper focuses on a single proposed mechanism without ruling out alternatives, consider further qualifying the claim (e.g., specifying 'in part' or 'under certain conditions').*

We thank the reviewer for this suggestion. As described in our response to Major Comment 1, we have revised the title to remove the mechanistic claim entirely, so that it no longer references the proposed spectral-bandwidth mechanism at all and now reads as "Persistent residual ellipticity in waveplate-compensated polarization-resolved SHG microscopy on a commercial multiphoton microscope." We believe this addresses the reviewer's underlying concern more directly than further qualifying language would.

2. *Waveplate ordering clarification (p. 2): The practical implications of the QWP-HWP vs. HWP-QWP ordering difference for the compensation mathematics should be stated explicitly in the main text rather than relegated to the Acknowledgments, as it is methodologically relevant.*

We thank the reviewer for this suggestion, which we agree with. We have added an explicit discussion of the waveplate ordering and of its practical implications to the Materials and Methods Section as follows:

"This waveplate compensation approach was originally developed by Chou et al. using a HWP-QWP configuration. The waveplate ordering is critical as it determines the mathematical formulation of the compensation curves. The QWP-HWP order offers the practical advantage that the QWP can be fixed while HWP rotation controls the polarization angle."

We have also added a corresponding note in the Results Section, where the Jones matrix simulation is described, clarifying that we adopted the QWP-HWP ordering of Romijn et al. rather than the HWP-QWP ordering of Chou et al.'s original optical model, and pointing back to the Methods Section for the practical rationale. The original acknowledgment to Elisabeth I. Romijn remains in place, but the substantive methodological content is now presented in the main text.

3. *Figure 2B: The caption states the light blue curve shows ellipticity reduced to zero, but the minimum and maximum ellipticity achieved with this configuration should be stated explicitly as numerical values.*

We thank the reviewer for this comment. In addressing it, we reviewed how ellipticity was extracted in our OpticStudio simulations. In the original submission, ellipticity was computed directly in OpticStudio using the CODA merit function operand to extract the polarization ellipse parameters, with an internal optimization determining the compensation curve. In this revision, we replaced this approach with one that more closely mirrors our experimental measurement: an ideal linear polarizer is added at the end of the simulated optical path and rotated over 360°, and the resulting transmitted intensity curve is fit using the same procedure applied to experimental data (Equation 7 from Romijn et al.), rather than extracting ellipticity directly from OpticStudio's internal polarization-ellipse calculation. This revised methodology is now described in the new Appendix S5.

As part of this revision, we also restructured Figure 2 to focus solely on the HWP-only measurements and on their simulation and moved the compensation-curve comparison (including the light blue curve referenced in the comment) to an expanded, new Figure 4, which presents this comparison across four conditions (monochromatic/polychromatic, ideal/real waveplate retardance) using the revised extraction method. The corresponding minimum- and maximum-ellipticity values for each condition are now stated explicitly in the text accompanying Figure 4. For example, with real waveplate retardance under monochromatic illumination, the minimum-ellipticity curve remains below 0.01 while the curve obtained by fitting the model of Romijn et al. reaches a maximum of approximately 0.05; combining real retardance with polychromatic illumination, this fitted curve reaches a maximum of approximately 0.11 and a minimum of approximately 0.017, addressing the reviewer's request.

4. *Figure 3B: The legend includes 'HWP+QWP*' but the caption does not explain what the asterisk denotes. This should be clarified.*

We thank the reviewer for this comment. The asterisk was intended to flag that two solutions exist for the compensation curve at each HWP angle, but we agree the original figure did not make this sufficiently clear. We have revised Fig 3 to improve the visual distinction between the two solutions: in panel A (sample-plane map), we added annotations "(1)" and "(2)" directly next to the two dashed compensation curves. In panel B, we revised the line styles and colors: the HWP-only curve is now a semi-transparent solid black line, and the two compensation solutions are shown as a dotted orange line (solution 1) and a dashed blue line (solution 2). The vertical lines marking the HWP motor zero position for each curve use the corresponding line style (with added transparency). Because the two solutions overlap substantially, we believe these changes make them easier to distinguish while preserving the visibility of their close agreement.

5. *Figure 4D: The monochromatic curve shows small oscillations near zero that appear to be numerical fitting artifacts. Their origin should be acknowledged and explained.*

We thank the reviewer for this observation. Following a substantial revision of Figure 4 in response to other comments (see our responses to Major Comments 1, 2, and 7), the figure now comprises eight panels covering four conditions (monochromatic and polychromatic illumination, each with ideal and real waveplate retardances). The small oscillations noted by the reviewer correspond to the minimum-ellipticity curve for the monochromatic, ideal-waveplate condition (new Figure 4A-B), which we now explicitly address in the main text as follows:

"The beam is therefore essentially linearly polarized across the full range of polarization angles, with the small residual noise in the minimum-ellipticity curve likely reflecting the discretization of the map rather than a real effect."

This curve is obtained by taking the minimum ellipticity directly from a simulated map sampled at discrete HWP and QWP angles, rather than from a continuous fit. The true minimum ellipticity at a given HWP angle may fall between sampled QWP angles, which we believe explains the small residual oscillations observed.

6. *Missing objective specification: The numerical aperture and model of the objective lens used in all measurements must be stated in the Materials and Methods section. This is a critical experimental parameter for interpreting polarization results.*

We thank the reviewer for this comment. As detailed in our response to Major Comment 4, no objective lens was present in the beam path for any of the polarization measurements reported in this manuscript. Following the calibration procedures of Romijn et al. and Chou et al., the objective lens is removed, and polarization is measured at the sample plane. An objective NA and model are therefore not applicable to the calibration measurements presented here. We have revised the Materials and Methods Section to state this explicitly and unambiguously, so that it is clear no objective lens data is missing, but rather that none was used.

7. *Laser bandwidth discrepancy: A simulation at the actual specified bandwidth (12.5 nm FWHM) should be included alongside the 20 nm case, as it would provide a more direct comparison to the experimental system.*

We thank the reviewer for this comment, which we agree with. In this revision, we replaced the 20 nm bandwidth used in the original submission with the laser's actual specified bandwidth of 12.5 nm FWHM throughout the polychromatic simulation presented in the main text (Figure 4E-H), so that this is now the only bandwidth condition shown there, rather than an additional case alongside 20 nm. We have additionally added a dedicated bandwidth sensitivity analysis (new Supplementary Figure S9) directly

comparing monochromatic illumination to 2 nm, 12.5 nm, and 25 nm FWHM bandwidths at a fixed dichroic retardance-versus-wavelength slope, allowing a direct assessment of how the simulated residual ellipticity depends on bandwidth relative to our system's actual specification.

8. *Detection sensitivity at low ellipticity (p. 4): Since the entrance port measurements show ellipticity values approaching the photodiode's ~0.074 reliability threshold, this limitation should be discussed more prominently in relation to the entrance port characterization.*

We thank the reviewer for this comment. We would like to clarify that the 0.074 threshold reported in Supplementary Figure S4 marks the point above which agreement between the photodiode and power meter is excellent (differences below 0.003), not a hard cutoff below which measurements become unreliable. Below this threshold, the degradation is gradual rather than abrupt: at a power-meter-measured ellipticity of 0.022, comparable to the lower end of our entrance-port measurements, the photodiode reads a mean of approximately 0.017, an absolute error of about 0.005, with a measurement spread comparable to that of the power meter itself at the same condition. This is consistent with what is visible in our entrance-port data (Figure 2A): the curve becomes somewhat noisier at its lowest points but remains continuous and well-behaved, without any sign of an abrupt loss of measurement quality. Given that our entrance-port ellipticity values already fall within the laser's specified polarization extinction ratio (equivalent to an ellipticity of 0.045), and that the effects under investigation in this work (0.06 to 0.25 at the sample plane) extend well above this level, we do not believe that this modest, systematic underestimation materially affects our characterization of the entrance port as effectively linearly polarized, or our broader conclusions. We have, however, revised the relevant sentence in the main text to note explicitly that entrance-port values lie near or below this threshold and that the photodiode may modestly underestimate ellipticity in this range.

9. *S1 Table parameter discrepancy: The fitted HWP angle theta_0 varies by 7.5 deg between measurement locations. This discrepancy should be discussed more carefully, as it may indicate that the Romijn et al. model is more substantially misspecified than the text implies.*

We thank the reviewer for flagging this discrepancy, which prompted a substantial addition to this revision. We agree that this variation warranted careful investigation rather than a passing mention, and we have added the following analyses to the manuscript.

First, we confirmed that this discrepancy is reproducible: we repeated the full map acquisition and fitting procedure on two additional, independent days (new Table 3, Exp 2 and 3), and the same entrance-port/sample-plane discrepancy in the fitted HWP angle

(as well as the polarization analyzer angle) persisted in every experiment, while the QWP angle remained stable throughout.

Second, we identified a specific, quantifiable mechanism contributing to this discrepancy. Using our monochromatic Jones matrix simulation, we generated synthetic ellipticity maps with known ground-truth waveplate and analyzer angles and fitted them with the model of Romijn et al., comparing ideal and manufacturer-specified (real) waveplate retardances at system retardances comparable to our experimental entrance-port and sample-plane values (new Supplementary Table S1). This showed that the QWP angle is robustly recovered regardless of waveplate retardance, whereas introducing real retardance substantially degrades the accuracy of the recovered HWP and analyzer angles, with larger errors at the lower system retardance characteristic of the entrance port.

Third, we investigated this pattern analytically. We found that in the limiting case of zero system retardance, the model of Romijn et al. is genuinely non-identifiable in these two parameters: the HWP and polarization analyzer angles cannot be individually recovered from fitting, only a specific combination of the two can (new Appendix S4). This degeneracy is consistent with the larger fitting errors observed at lower system retardance in Supplementary Table S1, though we have not characterized how the error scales with retardance away from this limiting case.

We agree with the reviewer that this reflects a genuine limitation of the Romijn et al. model rather than a minor discrepancy, and we have revised the Results and Conclusions Sections to state this explicitly. We note, however, that this specific limitation concerns the model's ability to individually resolve the fitted HWP and analyzer angles ($\theta_0$, $\alpha_0$) at low system retardance; the linear combination of these two parameters that determines the optimal compensation curve remains identifiable, so the derivation of the compensation curve itself is not affected by this degeneracy. This is a distinct issue from the substantial residual ellipticity we report even along the optimal compensation curve, which is the central finding of this work and which we attribute, as discussed throughout the manuscript, to the polychromatic mechanism rather than to a failure to correctly identify the compensation angles.

10. *AI assistance disclosure: The authors note use of Claude 4.5 Sonnet for manuscript preparation. PLOS ONE's editorial policies on AI use should be verified to ensure full compliance with the journal's current requirements.*

We thank the reviewer for this comment. This disclosure was in fact motivated by a careful review of PLOS's current policy on the use of AI tools in manuscript preparation, and we confirm that our disclosure is consistent with its three requirements: it identifies the specific tool used (Claude Sonnet 4.5, Anthropic), describes how it was used (structuring arguments, refining technical descriptions, improving clarity) and how its outputs were evaluated (all AI-assisted text was reviewed and edited by the authors), and states that the authors retain full responsibility for the scientific content. We have

updated the disclosure to reflect the specific Claude Sonnet version used in this revision (Claude Sonnet 5). Regarding placement, we currently retain this disclosure in the Acknowledgments, which we understand to be an acceptable location under PLOS's policy. We defer to the editor's guidance should a different placement be preferred.

11. *Equation self-containedness: Equations 1-3 from Romijn et al. are used without re-deriving or restating them. At minimum, the physical meaning of each parameter in those equations should be briefly defined in the text for the paper to be self-contained.*

We thank the reviewer for this comment. Since our study is intended as a direct extension of Romijn et al. using their established calibration method and model, we expect readers interested in the details of this fitting procedure to be familiar with, or to minimally consult, the original publication introducing it. Consequently, we did not consider it necessary to fully re-derive their equations here. That said, we agree that all parameters should be clearly defined in our own text, which is already the case as the physical meaning of each parameter in Equations 1-3 ($I_0$, γ, δ, $\theta_0$, $\phi_0$, and $\alpha_0$) is defined in the caption of Table 3. In the original submission, this table was included as a supplementary item. In the revision, however, in response to other comments (see our response to Major Comment 5), we have moved it into the main text. These parameter definitions thus now immediately appear in the main manuscript and do not require a consultation of the supplementary material.

12. *References: Reference 6 (Cisek et al., 2023) is a book chapter that may be difficult to access. Where possible, peer-reviewed journal articles should be prioritized for core methodological citations.*

We thank the reviewer for this comment. We note that this citation supports a general, introductory description of the standard polarization-resolved SHG measurement approach, rather than a core methodological citation specific to the compensation method evaluated in this study, which is drawn entirely from peer-reviewed journal articles (Romijn et al. and Chou et al.). That said, we agree that accessibility is a reasonable concern, and we have therefore added an additional, freely accessible peer-reviewed reference alongside the existing citation: Brasselet, S. (2011), "Polarization-resolved nonlinear microscopy: application to structural molecular and biological imaging," Advances in Optics and Photonics, 3(3), 205-271, a widely cited tutorial review on this topic published in an Optica journal.

# Reviewer 3

## Major Comment 1

*First, the novelty of the work should be more clearly articulated. While the study is well executed, polarization distortions and wavelength-dependent*

> *birefringence in optical systems are already known. The authors should explicitly define what new insight is provided beyond prior work (e.g., Romijn et al.), particularly whether the main contribution lies in identifying the magnitude of residual ellipticity, demonstrating the limitations of compensation, or linking the effect to femtosecond laser bandwidth.*

We thank the reviewer for this comment. We would like to note that PLOS ONE's editorial criteria explicitly state that publication decisions are made on the basis of scientific and technical rigor, and do not require novelty or anticipated impact. This is in fact what motivated our choice for this venue, as it is intended, among other things, as a home for negative results and reproducibility studies. Given this, we have chosen not to reframe the manuscript around a novelty claim it was not designed to make. Rather than proposing a new phenomenon, this study is, at its core, an independent reproducibility test of an established method (Romijn et al.) on the same class of commercial instrument used to develop it, reporting that it provided minimal practical benefit in our system. The proposed mechanistic hypothesis is offered as a plausible, but explicitly unconfirmed, explanation for this result, not as the paper's central claim.

That said, we agree that the specific, concrete findings of the study were not stated clearly enough, and we have revised the Introduction to state them explicitly: (1) an independent reproduction of the Romijn et al. compensation method showing minimal practical benefit under our system's specific (low) system retardance; (2) an identified and analytically confirmed limitation of the underlying fitting model itself, wherein two of its parameters become non-identifiable at low system retardance (new Supplementary Table S1 and Appendix S4); and (3) a quantitatively bounded, though unconfirmed, hypothesis for a possible physical origin of the residual pattern, supported by a systematic sensitivity analysis (new Supplementary Figures S7-S9).

## Major Comment 2

> *Second, the central conclusion—that residual ellipticity arises from the combination of spectral bandwidth and wavelength-dependent dichroic retardance—is supported primarily by simulations and qualitative agreement with experiments. There is no direct experimental validation of this hypothesis. The authors are encouraged to either (i) provide additional experimental evidence (e.g., bandwidth filtering or comparison with a narrower linewidth source), or (ii) moderate the strength of their claims and clearly frame this as a plausible explanation rather than a definitive conclusion.*

We thank the reviewer for this comment, which echoes a concern also raised by Reviewer 2 (Major Comment 1), and we have adopted the second of the reviewer's suggested options. We have substantially moderated the language throughout the manuscript, including modifying the title, abstract, results, and conclusions, so that the

spectral-bandwidth mechanism is consistently presented as a proposed, unconfirmed explanation (e.g., "may arise from," "we hypothesize," "a possible, though not fully verified, explanation") rather than a definitive conclusion. The new title no longer references this mechanism at all.

We have also added a quantitative sensitivity analysis (new Supplementary Figures S7-S9) assessing how strongly our modeling assumptions would need to be pushed to reproduce the full experimental residual ellipticity amplitude, and we explicitly state in the discussion that this required an unusually large retardance span that we consider unlikely to be representative of our actual, undocumented dichroic mirror. We believe this analysis represents a meaningful, if partial, step toward the reviewer's suggestion (i): rather than a direct experimental validation (e.g., bandwidth filtering or a narrower-linewidth source, both of which we agree would provide a more definitive test but are not feasible in the context of this study), it establishes quantitative bounds on the plausibility of the mechanism using our existing simulation framework.

We agree that bandwidth filtering or a comparison against a picosecond source would provide more direct experimental validation, and we have stated explicitly in the discussion that we were unable to perform either within the scope of this study. The dichroic mirror is indeed in continuous use on a shared facility instrument and cannot not be removed for ellipsometry, and we do not have access to a suitable narrow-linewidth source at the relevant wavelength. We have framed these as necessary next steps for confirming the proposed mechanism, consistent with the more cautious framing adopted throughout the revised manuscript.

## Major Comment 3

> *Third, the simulation model appears simplified (e.g., linear retardance assumption, limited spectral sampling), and does not fully reproduce the experimentally observed amplitude of ellipticity. A more critical discussion of these limitations, along with sensitivity analysis or justification of modeling assumptions, would strengthen the manuscript.*

We thank the reviewer for this comment, which overlaps with concerns raised by Reviewer 2 (Major Comments 2 and 3). We hereafter address both the linear retardance assumption and the spectral sampling in turn.

Regarding the linear retardance assumption, we agree that the true wavelength dependence of our dichroic mirror's retardance is unknown and could, in principle, be nonlinear or non-monotonic. Rather than testing an arbitrary nonlinear profile, which would not represent a validated alternative, we instead added a systematic sensitivity analysis varying the total span of a linear retardance profile (new Supplementary Figure S8) and the illumination bandwidth (new Supplementary Figure S9), which we believe conveys more transparently the uncertainty of the model. This analysis shows that even

the most extreme span tested (±60°, 120° total) reaches only a simulated amplitude of approximately 0.22, approaching but not fully reproducing the experimental maximum of approximately 0.24 (as noted in our response to Reviewer 2's Major Comment 2, we use this more precise experimental value here for quantitative comparisons), which we consider unlikely to be representative of our specific, undocumented dichroic mirror. We have revised the discussion to state this limitation explicitly.

Regarding spectral sampling: we added a dedicated convergence analysis (new Supplementary Figure S7), comparing coarser wavelength samplings against a 31-wavelength reference simulation. This confirmed that the 15 wavelengths used throughout the revised manuscript (increased from 9 in the original submission, to be conservative) closely approximate the finely sampled reference, and we now state this justification explicitly at the first mention of the wavelength sampling in the main text.

We have also revised the discussion to more critically and explicitly acknowledge the remaining sources of uncertainty in the model: our assumption of a linear retardance-versus-wavelength dependence, uncertainty in the actual retardance spectrum of our specific dichroic mirror, and the possibility of additional, undocumented wavelength-dependent optical elements in our closed, commercial system that are not captured by the model.

## Major Comment 4

> *Fourth, the study is conducted on a single microscope system. The authors should discuss the generalizability of their findings to other systems and optical configurations, and clarify whether the observed limitations are expected to be universal or system-dependent.*

We thank the reviewer for this comment and agree that this distinction was not made explicit enough in the original submission. We believe it is useful to separate two aspects of our findings that differ in their generalizability.

We first would like to emphasize that the system we consider was not arbitrarily chosen: our study reproduces an established compensation method (Romijn et al.) using the same class of commercial instrument (Leica TCS SP8 MP) the method was originally developped and validated on. We are not proposing a new method on a novel system, but testing the practical performance of an existing, published method on the same instrument type it was designed for. In this sense, our finding that the method provides minimal practical improvement is directly relevant to any scientist using or considering this specific method on this specific class of commercial microscope, which we believe is already a meaningful scope.

Beyond this specific instrument class, we agree it is useful to separate two aspects of our findings that differ in their broader generalizability. The specific magnitude of

residual ellipticity we report (up to 0.24) and the specific system retardance underlying it (12.1° at the sample plane) are, in principle, properties of our particular instrument and would not necessarily be expected to generalize numerically to other microscope models or optical designs. We note, however, that a comparable magnitude of residual ellipticity and a similar periodic pattern are also present, though not extensively discussed, in the originally published data of Romijn et al. (their Figure 6b), obtained on the same class of commercial instrument as ours. This suggests that, at least among similar Leica TCS SP8 MP-type systems, the magnitude of this effect may be more consistent than would be expected from an arbitrary property of a single instrument, though we do not have data from other microscope models to test this further. We already note in the Conclusions Section that our system exhibited a relatively modest system retardance, and that systems with substantially higher birefringence may see more dramatic absolute improvement from waveplate compensation, even though we would still expect the underlying residual ellipticity variation to persist. We have now expanded this discussion to state these points more explicitly.

In contrast, we believe that the underlying physical mechanisms we investigate are not specific to our system and can be expected to generalize, at least qualitatively, to other femtosecond multiphoton systems using similar optical designs, whether or not they use the same commercial platform. Differential reflection phase shifts at metallic mirrors, and wavelength-dependent retardance in dichroic mirror coatings, are general properties of these optical elements, documented independently of our specific instrument (e.g., Schön et al., for a different dichroic mirror than ours). Any waveplate-compensated system combining a broadband femtosecond source with wavelength-dependent optical elements would, in principle, be subject to the same qualitative limitation we describe: compensation optimized for a single wavelength cannot simultaneously compensate all spectral components of a broadband pulse. Whether this limitation is practically significant for a given system depends on the magnitude of that system's wavelength-dependent retardance relative to its spectral bandwidth, which is expected to vary substantially between instruments and cannot be predicted from our single-system study.

We have revised the Discussion and Conclusions Sections to state this distinction explicitly.

## Major Comment 5

> *Fifth, the practical impact of the reported ellipticity values (up to 0.25) is not quantitatively analyzed. Since polarization-resolved SHG is often used for structural inference, it would be valuable to include a clearer discussion—or preferably a quantitative example—of how such residual ellipticity affects downstream measurements or interpretation.*

We thank the reviewer for this comment, which overlaps with a related concern raised by Reviewer 2 (Major Comment 6). We agree that a quantitative link between residual ellipticity and errors in downstream structural parameters would be valuable, but this would require dedicated analysis and validation efforts that falls outside the scope of the present study, which is focused on characterizing the polarization state itself rather than its propagation into structural inference. We have revised the manuscript to state this limitation explicitly rather than speculating about acceptable ellipticity thresholds without quantitative support:

"Because ellipticity varies with polarization angle, it could in principle affect the shape of polarization-dependent SHG intensity polar plots, which are commonly used to infer sample orientation and microstructural organization. As a quantitative assessment of this effect on downstream analysis is beyond the scope of the present study, we restricted our observations to the polarization state itself."

We believe this appropriately scopes our central finding, namely that waveplate compensation provided minimal practical improvement in our system, independently of any specific downstream-error threshold, while clearly flagging the open question the reviewer raises as an important direction for future work.

## Major Comment 6

> *Finally, while the manuscript is generally well written, some sections (particularly in Materials and Methods) are overly detailed and could be streamlined or moved to supplementary materials to improve readability.*

We thank the reviewer for this comment. As noted in our response to Major Comment 1, we consider this work primarily a reproducibility study: we implemented a previously published compensation method on a different but comparable commercial system and report that it did not yield the expected improvement. Given this framing, we felt it was important to document our experimental implementation in sufficient detail that other groups could reproduce, verify, or build on it directly, including practical details (e.g., hardware components, acquisition timing, DAQ configuration) that a more conventional results-driven paper might omit or relegate to supplementary material. We have considered the reviewer's point carefully, but on balance believe the current level of detail serves the reproducibility goals of the work, and we would prefer to retain it. We remain happy to trim specific passages if the editor considers this necessary for readability.

# Reviewer 4

## Major Comment 1

> *First, the core hypothesis lacks direct experimental data support. The authors claim that residual ellipticity originates from the combination of the wavelength-dependent retardance of the dichroic mirror and the broadband spectrum of the laser. However, they explicitly admit to not measuring the actual retardance spectrum of the Leica microscope dichroic mirror used, instead directly inferring it from referenced data. This unverified substitution weakens the direct persuasiveness of the conclusions.*

We thank the reviewer for this comment, which echoes closely related concerns raised by Reviewer 2 (Major Comment 1) and Reviewer 3 (Major Comment 2). Our proposed mechanism is indeed supported by simulation using literature-derived retardance values rather than by direct measurement of our own dichroic mirror, and we have substantially revised the manuscript to reflect this limitation more clearly.

Specifically, we have moderated the language throughout the manuscript, including the title, abstract, results, and conclusions, so that the spectral-bandwidth mechanism is consistently presented as a proposed, unconfirmed explanation (e.g., "may arise from," "we hypothesize," "a possible, though not fully verified, explanation") rather than a demonstrated conclusion. The new title no longer references this mechanism at all. We have also added a systematic sensitivity analysis (new Supplementary Figures S7-S9) to assess how sensitive our conclusions are to this substitution: varying the assumed span of dichroic retardance across the illumination bandwidth shows that reproducing the full experimental residual ellipticity amplitude would require an unusually large retardance span that we consider unlikely to be representative of our specific, undocumented dichroic mirror, and we now state this limitation explicitly in the discussion.

We agree that direct measurement of our dichroic mirror's retardance spectrum (e.g., by ellipsometry) would be required to fully confirm the proposed mechanism, and we state this now as an important direction for future work. We were unable to perform this measurement within the present study because the dichroic mirror is continuously used on a shared facility instrument and could not be removed for ellipsometry.

## Major Comment 2

> *Second, there is a significant quantitative discrepancy between the Jones matrix simulation results and the experimental data. The residual ellipticity amplitude derived from the polychromatic light simulation is only around 0.13, whereas the actual experimental measurement is as high as 0.25. Although the authors mention this difference, they fail to further refine the physical model to bridge*

*this gap, indicating that the current theoretical explanation is quantitatively incomplete.*

We thank the reviewer for this comment, which raises the same concern as Reviewer 2 (Major Comment 2). We agree that the original submission did not adequately investigate this quantitative gap, and we have added a systematic parametric sensitivity analysis to the manuscript (new Supplementary Figures S8 and S9) to assess it directly.

Specifically, we varied (1) the total span of dichroic retardance across the illumination bandwidth, from 0° up to ±60° in steps of 10° (Supplementary Figure S8), and (2) the illumination bandwidth itself (monochromatic, 2 nm, 12.5 nm, and 25 nm FWHM), at a fixed retardance-versus-wavelength slope (Supplementary Figure S9). This analysis shows that even the most extreme span tested (±60°, i.e. 120° total) over our laser's 12.5 nm bandwidth reaches only a simulated amplitude of approximately 0.22, approaching but not fully reproducing the experimental maximum of approximately 0.24 (as noted in our response to Reviewer 2's Major Comment 2, we use this more precise experimental value here for quantitative comparisons). More moderate, arguably more plausible spans produce substantially smaller residual ellipticity that are consistent with the gap the reviewer identifies. We have revised the discussion to state this limitation explicitly.

We agree with the reviewer that this indicates that the current model is quantitatively incomplete, and we do not believe this gap can be convincingly closed by further refining the model with additional free parameters we cannot independently justify or verify against our specific instrument. Doing so would risk simply fitting the discrepancy away rather than explaining it. We have instead revised the discussion to explicitly enumerate the candidate sources of this remaining discrepancy: our simplified assumption of a linear dependence between dichroic retardance and wavelength, uncertainty in the actual retardance spectrum of our specific dichroic mirror, and the possibility of additional, undocumented wavelength-dependent optical elements in our closed, commercial system that are not captured by the model. We regard closing this quantitative gap, most directly through ellipsometric characterization of our dichroic mirror, as an important direction for future work.

## Major Comment 3

*Third, the parameter settings for the theoretical simulations are disconnected from the actual experimental equipment. The Mai Tai femtosecond laser used in the experiment has a nominal bandwidth of 12.5 nm, yet the authors employed a bandwidth of 20 nm in the simulation. The practice of artificially relaxing parameters to highlight spectral effects lacks rigor. Furthermore, even with the expanded bandwidth parameter, the simulated polarization distortion remains far below the true experimental value.*

We thank the reviewer for this comment, which echoes a comment from Reviewer 2 (Minor Comment 7). In the revision, we replaced the 20 nm bandwidth used in the original submission with the laser's actual specified bandwidth of 12.5 nm FWHM throughout the polychromatic simulation presented in the main text, so that all polychromatic results now reflect our actual experimental laser specification rather than an artificially widened parameter.

We would also like to note, for transparency, that the dichroic retardance span used together with this bandwidth was not inflated to manufacture agreement with the experimental amplitude: Schön et al., our source for the general magnitude of dichroic dispersion, report variations exceeding 50° over comparable spectral ranges, and our original span (−12.9° to +37.1°, 50° total) was comparable to, not larger than, this reported magnitude. In the revised manuscript, our reference condition uses a ±30° span (60° total), paired with the correct 12.5 nm bandwidth. We have also added a dedicated retardance-span sensitivity analysis (new Supplementary Figure S8) testing spans up to ±60° (120° total) to show how far this parameter would need to be pushed to approach the experimental amplitude, and to allow the reviewer and future readers to judge this choice directly.

We agree that using an inflated bandwidth to more closely approach the experimental amplitude, rather than because it reflects the real system, would not have been a rigorous approach, and we did not intend to represent it as more than an initial approximation using a round-number placeholder. We now instead directly test how much the results depend on this choice: we have added a dedicated bandwidth sensitivity analysis (new Supplementary Figure S9) comparing monochromatic illumination to 2 nm, 12.5 nm, and 25 nm FWHM bandwidths at a fixed dichroic retardance-versus-wavelength slope, so that the relationship between bandwidth and residual ellipticity, and the specific result at our laser's actual specification, are both stated transparently and quantitatively.

Regarding the remaining quantitative gap even at our actual bandwidth: we agree this indicates that the mechanism, as currently modeled, is not quantitatively sufficient on its own, and we discuss this directly, together with a corresponding retardance-span sensitivity analysis (new Supplementary Figure S8), in our response to the reviewer's Major Comment 2 above.

## Major Comment 4

> *Fourth, the established optical system model is overly simplified and ignores key optical components. The simulation only includes waveplates, five metal mirrors, and a dichroic mirror, completely omitting the high numerical aperture objective lens. High numerical aperture objectives are known to cause severe polarization distortions. Excluding the most critical imaging component of the*

*microscope results in a simulation environment that fails to reflect the complexity of the optical path at the sample plane.*

We thank the reviewer for this comment, and we apologize that this was not stated clearly enough in our original submission. As explained in our response to Reviewer 2 (Major Comment 4), no objective lens was present in the beam path for any of the experimental measurements reported in this manuscript. As in the calibration procedures of Romijn et al. and Chou et al., the two studies whose method and optical model we build directly upon, the objective lens was removed and polarization measured at the sample plane (i.e., the position where the sample would normally be located during imaging). This is not a simplification we introduced, but the established convention of the specific compensation method we are reproducing and of its associated calibration procedure. Our simulation models this same, deliberately objective-free configuration.

We fully agree that high-NA objectives are known to introduce significant polarization distortions, and that a complete characterization of the polarization state actually delivered to a specimen during imaging would need to include the objective's contribution. This is precisely why the calibration procedure we, and the prior work we build on, follow removes the objective: it isolates the contribution of the upstream optics (waveplates, mirrors, dichroic mirror) from that of the objective, which is a substantial, separate, and well-studied topic in its own right. We have revised the Materials and Methods, Results, and Conclusions Sections to state this explicitly and unambiguously: the present study characterizes the polarization state delivered to the objective, and does not address objective-induced depolarization, which we now flag as a further consideration for full-system polarization accuracy in high-NA imaging, citing the relevant literature.

## Major Comment 5

*Fifth, there are issues with the applicability of the fitting model, resulting in fitting parameters that lack a reasonable physical basis. According to the supplementary data, the initial angle parameter of the half-wave plate exhibits a massive shift from 18.07 degrees to 25.61 degrees between the entrance port and the sample plane. Since the physical mounting position of the half-wave plate remained unchanged, this parameter jump is illogical. The authors simply attribute this to the model's inability to fully capture the sources of residual ellipticity, exposing a lack of robustness in their mathematical fitting model when handling real system errors.*

We thank the reviewer for highlighting this discrepancy, which prompted a substantial investigation in this revision as mentioned already in response to Reviewer 2 (Minor Comment 9). While we cannot claim to have fully resolved it, we no longer believe it

should be characterized as illogical or unexplained as we have identified a specific, mathematically grounded contributing mechanism.

First, we confirmed that this discrepancy is reproducible: we repeated the full map acquisition and fitting procedure on two additional, independent days (new Table 3, Exp 2 and 3), and the same entrance-port/sample-plane discrepancy in the fitted HWP angle persisted in every experiment, while the QWP angle (whose physical mounting was also unchanged) remained stable throughout, as expected.

Second, using our monochromatic Jones matrix simulation, we generated synthetic ellipticity maps with known ground-truth waveplate and analyzer angles and fitted them with the model of Romijn et al., comparing ideal and manufacturer-specified (real) waveplate retardances at system retardances that were comparable to our experimental entrance-port and sample-plane values (new Supplementary Table S1). Because the ground-truth angles are known in this simulated case, we could directly quantify the fitting error, and found that the QWP angle was robustly recovered regardless of waveplate retardance, whereas the introduction of real, non-ideal retardance substantially degraded the accuracy of the recovered HWP and analyzer angles specifically, with larger errors at the lower of the two system retardances tested.

Third, this pattern led us to examine a specific limiting case analytically. We derived that, for a representative case (zero system retardance, unit reflection ratio, consistent with the values we obtain from fitting our own experimental data), the model of Romijn et al. is genuinely non-identifiable in exactly these two parameters: the HWP and polarization analyzer angles cannot be individually recovered from fitting, only a specific linear combination of the two can (new Appendix S4). We note that this derivation is limited to this representative case. We have not derived the general behavior for arbitrary reflection ratios, and this result should be understood as demonstrating that this degeneracy exists and is relevant to our system, not as a complete analytical theory of the model's behavior at all retardances.

Taken together, we believe the entrance-port/sample-plane discrepancy we observe experimentally is consistent with, and plausibly explained by, this identifiability limitation, which our simulation shows becomes more severe as system retardance decreases. We have revised the manuscript to state this more carefully, presenting the mechanism as a plausible, mathematically supported explanation for the discrepancy.

# Journal Requirements

## Editorial Comment 1

> *Please ensure that your manuscript meets PLOS ONE's style requirements*

We thank the editorial staff for this note. We have reviewed the manuscript against the PLOS ONE formatting templates and made the following corrections: (1) the Supporting information section, which was previously placed before the references, has been moved to follow the reference list, consistent with the required section order (main text, Acknowledgments, References, Supporting information); this also addresses point 4 below regarding the placement of Supporting Information legends relative to the reference list. (2) Two instances where figures were cited as "Figure" rather than the required "Fig" have been corrected. We have otherwise confirmed that the manuscript's title, abstract length, Supporting Information numbering, and figure/table/equation citation formats comply with the PLOS ONE style guidelines.

## Editorial Comment 2

> *ensure that your code is shared in a way that follows best practice and facilitates reproducibility and reuse.*

We thank the editorial staff for this note. We have reviewed PLOS ONE's code-sharing guidelines and, in this revision, archived both author-generated code repositories underlying the manuscript's findings (S2 File: data acquisition and analysis code; S3 File: OpticStudio simulation and automation code) on Zenodo, in addition to their existing GitHub repositories. Each Zenodo archive provides a permanent, versioned snapshot with a DOI, ensuring the code remains freely and persistently available without restriction, consistent with PLOS ONE's guidance on reproducibility and reuse.

## Editorial Comment 3

> *financial disclosure:*

We thank the editorial staff for this note. The funders had no role in study design, data collection and analysis, decision to publish, or preparation of the manuscript.

## Editorial Comment 4

> *supplementary table is included in the manuscript file. Please remove and upload it with the file type 'Supporting Information'*

We thank the editorial staff for this note. We have removed the S1 Table content from the manuscript file and uploaded it as a separate Supporting Information file. The corresponding legend remains in the manuscript's Supporting information section, after the references list.

## Editorial Comment 5

> *We note that Figure S1 in your submission contain copyrighted images.*

We would like to clarify that Supplementary Figure S1 is an original photograph we took of our own laboratory setup, showing the compensation module as installed on our

Leica TCS SP8 MP microscope. We (the authors) hold full copyright to this image, which was not sourced from, or reproduced from, any third-party publication or copyrighted material. The Leica and Thorlabs branding visible in the photograph are incidental and reflects the actual commercial equipment used in this study. It is not itself the subject of any copyright claim by the manufacturers over our photograph. We are therefore able to publish this image under the CC BY 4.0 license as required, and no third-party permission is needed.
