# Supplementary material for: Persistent residual ellipticity in waveplate-compensated polarization-resolved SHG microscopy on a commercial multiphoton microscope
Source: PLoS One. 2026 Nov 13;21(11):e0357256. doi: 10.1371/journal.pone.0357256 (PMC13766257; doi:10.1371/journal.pone.0357256)
Supplement: Supplementary file 13 [file pone.0357256.s023.pdf]

Decision Letter: Accept

Journal: PLOS ONE

Manuscript Number: PONE-D-26-06443R1

Full Title: Persistent residual ellipticity in waveplate-compensated polarization-resolved SHG microscopy on a commercial multiphoton microscope

Corresponding Author: Dr. David Nguyen

Dear Dr. Nguyen,

Thank you for submitting your revised manuscript and detailed Response to Reviewers for consideration by *PLOS ONE*.

I have carefully reviewed your revised manuscript, the track-changes version, and your point-by-point responses to the four reviewers. I am pleased to inform you that your manuscript is now **accepted for publication** in *PLOS ONE*.

Congratulations on this meticulous and open piece of work. Your findings will be of significant value to researchers working in nonlinear polarization optics and biomedical microscopy.

Sincerely,

Dr.邓

Academic Editor

PLOS ONE
