# Supplementary material for: Persistent residual ellipticity in waveplate-compensated polarization-resolved SHG microscopy on a commercial multiphoton microscope
Source: PLoS One. 2026 Nov 13;21(11):e0357256. doi: 10.1371/journal.pone.0357256 (PMC13766257; doi:10.1371/journal.pone.0357256)
Supplement: Supplementary file 14 [file pone.0357256.s020.docx]

**REVIEW REPORT**

| **Manuscript ID** | PONE-D-26-06443 |
| --- | --- |
| **Article Type** | Research Article |
| **Title** | Residual ellipticity in waveplate-compensated polarization-resolved SHG microscopy may arise from femtosecond laser spectral bandwidth |
| **Corresponding Author** | David Nguyen, ETH Zurich, Switzerland |
| **Review Date** | April 2026 |
| **Reviewer Expertise** | Polarization optics, nonlinear microscopy, biomedical SHG imaging |
| **Recommendation** | MAJOR REVISION |

# 1. Summary of the Manuscript

This manuscript investigates a fundamental limitation of waveplate-based polarization compensation in polarization-resolved second harmonic generation (pSHG) microscopy. The authors implement an established automated compensation method — a rotating half-waveplate (HWP) combined with a compensating quarter-waveplate (QWP) — on a commercial Leica TCS SP8 MP multiphoton microscope, building upon the approach of Romijn et al. (2018). Despite careful hardware implementation and significantly improved acquisition speed (360 ms per measurement vs. approximately 17 s in the original implementation), the authors consistently observe residual polarization ellipticity with amplitudes up to 0.25 that persists even under optimal waveplate configurations.

Through Jones matrix simulations incorporating polychromatic illumination, the authors propose that the residual ellipticity arises from the interaction between wavelength-dependent dichroic mirror birefringence and the broad spectral bandwidth (10-20 nm FWHM) of femtosecond laser pulses. Their polychromatic simulations qualitatively reproduce the observed periodic ellipticity variation (simulated amplitude ~0.13 vs. experimental ~0.25). The authors conclude with practical recommendations including specimen rotation or narrowband picosecond laser sources as alternatives for precision polarization control. Open-source analysis code, simulation files, and experimental datasets are provided.

# 2. Major Comments

The following major issues require substantive revision and should be addressed before the manuscript can be considered for acceptance.

**Major Comment 1: The Central Mechanistic Claim Lacks Direct Experimental Validation**

The paper's core hypothesis — that residual ellipticity arises from wavelength-dependent dichroic mirror birefringence combined with femtosecond pulse bandwidth — is supported only by simulation, not by direct measurement. The authors acknowledge that direct measurement of the dichroic mirror retardance spectrum 'would be required for quantitative validation but is beyond the scope of this work.' This is a significant limitation for a paper whose title asserts this mechanism as the explanation. The manuscript never actually measures the spectral retardance of the specific dichroic mirror in the Leica SP8 system; instead it borrows values from Schon et al. (2008), which were obtained on a different system. Without at least a qualitative experimental test — for example, comparing ellipticity residuals when using a narrowband (picosecond) versus broadband (femtosecond) source at the same center wavelength — the causal claim remains speculative. The authors should either perform this comparison, characterize their dichroic mirror directly using ellipsometry or the Mueller matrix method, or substantially moderate the language of their title and conclusions to reflect that the mechanism is proposed rather than demonstrated.

**Major Comment 2: Quantitative Mismatch Between Simulation and Experiment Is Insufficiently Addressed**

The polychromatic simulation produces residual ellipticity of approximately 0.13, while the experimental observations reach up to 0.25 — a factor of nearly two. While the authors list several possible contributing factors (simplified linear retardance model, uncertainty in the dichroic spectrum, other wavelength-dependent elements), no quantitative sensitivity analysis is performed to determine which factor is dominant or whether the proposed mechanism is even sufficient to account for the observed magnitude. This discrepancy is non-trivial: it opens the possibility that other unaccounted mechanisms — e.g., objective lens polarization aberrations, scan mirror retardance, polarization-dependent transmission in optical coatings — may contribute comparably or even dominantly. A parametric analysis showing how the simulated ellipticity amplitude changes with dichroic retardance slope, bandwidth, or the addition of further optical elements would substantially strengthen the paper's argument.

**Major Comment 3: The Simulation Model Makes Unverified Simplifying Assumptions**

The polychromatic Jones matrix simulation models the dichroic mirror retardance as varying linearly with wavelength from -12.9 deg to +37.1 deg over 40 nm. This linearity assumption is never justified. The reference measurement from Schon et al. (their Figure 6b) shows that dichroic retardance can exhibit strongly nonlinear, even oscillatory, spectral dependence depending on coating design. The actual spectral retardance of the Leica SP8 dichroic mirror is unknown. Furthermore, the simulation uses nine discrete wavelengths with a Gaussian weighting to represent the femtosecond pulse spectrum, but no justification is given for the adequacy of this sampling. The authors should: (1) test sensitivity of the simulation output to the retardance profile shape (linear vs. nonlinear), (2) verify convergence of the ellipticity estimate with respect to number of spectral samples, and (3) explicitly acknowledge that the borrowed dichroic values may not represent their instrument.

**Major Comment 4: The Role of Objective Lens Polarization Distortions Is Dismissed Too Quickly**

The authors do not specify the objective NA used, nor do they provide any quantification of its contribution to the observed ellipticity. Given that the paper attributes residual ellipticity specifically to the dichroic-bandwidth interaction, the absence of a systematic assessment of the objective lens contribution — particularly since measurements at the entrance port (ellipticity < 0.05) vs. sample plane (up to 0.25) show a significant increase through the microscope body — weakens the attribution. The authors should at minimum report the objective NA, reference appropriate literature on objective-induced polarization effects, and discuss whether this contribution has been ruled out or quantified.

**Major Comment 5: Statistical Characterization of Ellipticity Measurements Is Absent**

The paper presents ellipticity curves and maps as single-instance measurements without any statistical characterization — no error bars, no repeated measurement variability, no confidence intervals. The authors mention that their polarization analyzer takes 360 ms per measurement and that validation was done against a power meter (S4 Fig), but the reproducibility of the two-dimensional ellipticity maps (requiring 30 minutes of acquisition) is never assessed. Questions that remain unanswered include: How stable is the laser polarization state over 30 minutes? Does the ellipticity map change between acquisition runs? What is the measurement noise floor for the 2D maps? The authors should provide repeated measurements of at least the compensation curve (Fig. 3B) with appropriate error characterization, and discuss measurement stability over the full acquisition duration.

**Major Comment 6: The Practical Significance Threshold Is Not Adequately Justified**

The authors state that 'for some quantitative polarization-resolved measurements, a residual ellipticity of 0.25 may be acceptable depending on the required measurement precision.' This statement risks being misleading without context. No quantitative analysis is provided linking a given ellipticity value to resulting errors in downstream SHG analysis parameters (e.g., orientation angle error, molecular order parameter bias). The paper would be considerably strengthened by including either an analytical or numerical estimate of how ellipticity of 0.06-0.25 propagates into errors in the polarization-dependent SHG intensity ratio or fitted structural parameters. A worked example or sensitivity analysis connecting ellipticity magnitude to SHG measurement error is strongly recommended.

**Major Comment 7: Alternative Explanations for the Two-Peak Residual Pattern Are Not Ruled Out**

The two-peak periodic structure of the residual ellipticity along the compensation curve (Fig. 3B) is attributed to the polychromatic mechanism. However, this same two-peak structure could in principle arise from: (a) imperfect waveplate retardance calibration (the HWP is specified at 0.516 lambda instead of 0.5 lambda), (b) waveplate angular misalignment or wobble during rotation, (c) beam walk-off during HWP rotation, or (d) residual effects from the five silver mirrors not captured by the assumed model. The monochromatic simulation already shows that non-ideal waveplate retardances introduce asymmetry (Fig. 2B). The authors should explicitly demonstrate — through simulation or experimental controls — that these alternative explanations are insufficient to account for the observed residual ellipticity pattern before attributing it to the spectral bandwidth mechanism.

**Major Comment 8: The Proposed Alternative Solutions Are Insufficiently Analyzed**

The conclusions recommend specimen rotation and picosecond laser sources as alternatives, but neither is analyzed quantitatively in terms of its own practical limitations. Picosecond lasers reduce SHG efficiency (signal scales as peak power squared for pulsed excitation), which could be significant for weakly scattering biological specimens. The authors should provide at least a brief quantitative estimate of the SHG signal penalty expected when switching from femtosecond to picosecond pulses at the same average power, and discuss whether this tradeoff is acceptable for their targeted applications. Similarly, specimen rotation introduces translation-rotation coupling that the authors acknowledge but do not quantify in terms of required mechanical precision.

# 3. Minor Comments

The following minor issues should be addressed during revision:

- Title precision: The phrase 'may arise' is appropriately cautious, but given that the paper focuses on a single proposed mechanism without ruling out alternatives, consider further qualifying the claim (e.g., specifying 'in part' or 'under certain conditions').
- Waveplate ordering clarification (p. 2): The practical implications of the QWP-HWP vs. HWP-QWP ordering difference for the compensation mathematics should be stated explicitly in the main text rather than relegated to the Acknowledgments, as it is methodologically relevant.
- Figure 2B: The caption states the light blue curve shows ellipticity reduced to zero, but the minimum and maximum ellipticity achieved with this configuration should be stated explicitly as numerical values.
- Figure 3B: The legend includes 'HWP+QWP*' but the caption does not explain what the asterisk denotes. This should be clarified.
- Figure 4D: The monochromatic curve shows small oscillations near zero that appear to be numerical fitting artifacts. Their origin should be acknowledged and explained.
- Missing objective specification: The numerical aperture and model of the objective lens used in all measurements must be stated in the Materials and Methods section. This is a critical experimental parameter for interpreting polarization results.
- Laser bandwidth discrepancy: A simulation at the actual specified bandwidth (12.5 nm FWHM) should be included alongside the 20 nm case, as it would provide a more direct comparison to the experimental system.
- Detection sensitivity at low ellipticity (p. 4): Since the entrance port measurements show ellipticity values approaching the photodiode's ~0.074 reliability threshold, this limitation should be discussed more prominently in relation to the entrance port characterization.
- S1 Table parameter discrepancy: The fitted HWP angle theta_0 varies by 7.5 deg between measurement locations. This discrepancy should be discussed more carefully, as it may indicate that the Romijn et al. model is more substantially misspecified than the text implies.
- AI assistance disclosure: The authors note use of Claude 4.5 Sonnet for manuscript preparation. PLOS ONE's editorial policies on AI use should be verified to ensure full compliance with the journal's current requirements.
- Equation self-containedness: Equations 1-3 from Romijn et al. are used without re-deriving or restating them. At minimum, the physical meaning of each parameter in those equations should be briefly defined in the text for the paper to be self-contained.
- References: Reference 6 (Cisek et al., 2023) is a book chapter that may be difficult to access. Where possible, peer-reviewed journal articles should be prioritized for core methodological citations.
