# Supplementary material for: Persistent residual ellipticity in waveplate-compensated polarization-resolved SHG microscopy on a commercial multiphoton microscope
Source: PLoS One. 2026 Nov 13;21(11):e0357256. doi: 10.1371/journal.pone.0357256 (PMC13766257; doi:10.1371/journal.pone.0357256)
Supplement: Peer review history

# Peer review: Decision Letter 0

**DOI:** 10.1371/journal.pone.0357256.r001

**Authors:** Zeheng Wang

23 Apr 2026

Dear Dr. Nguyen,

Please submit your revised manuscript by Jun 07 2026 11:59PM. If you will need more time than this to complete your revisions, please reply to this message or contact the journal office at plosone@plos.org. When you're ready to submit your revision, log on to https://www.editorialmanager.com/pone/ and select the 'Submissions Needing Revision' folder to locate your manuscript file.

- A letter that responds to each point raised by the academic editor and reviewer(s). You should upload this letter as a separate file labeled 'Response to Reviewers'.
- A marked-up copy of your manuscript that highlights changes made to the original version. You should upload this as a separate file labeled 'Revised Manuscript with Track Changes'.
- An unmarked version of your revised paper without tracked changes. You should upload this as a separate file labeled 'Manuscript'.

If you would like to make changes to your financial disclosure, please include your updated statement in your cover letter. Guidelines for resubmitting your figure files are available below the reviewer comments at the end of this letter.

If applicable, we recommend that you deposit your laboratory protocols in protocols.io to enhance the reproducibility of your results. Protocols.io assigns your protocol its own identifier (DOI) so that it can be cited independently in the future. For instructions see: https://journals.plos.org/plosone/s/submission-guidelines#loc-laboratory-protocols. Additionally, PLOS ONE offers an option for publishing peer-reviewed Lab Protocol articles, which describe protocols hosted on protocols.io. Read more information on sharing protocols at https://plos.org/protocols?utm_medium=editorial-email&utm_source=authorletters&utm_campaign=protocols.

As the corresponding author, your ORCID iD is verified in the submission system and will appear in the published article. PLOS supports the use of ORCID, and we encourage all coauthors to register for an ORCID iD and use it as well. Please encourage your coauthors to verify their ORCID iD within the submission system before final acceptance, as unverified ORCID iDs will not appear in the published article. *Only*  the individual author can complete the verification step; PLOS staff *cannot*  verify ORCID iDs on behalf of authors.

We look forward to receiving your revised manuscript.

Kind regards,

Zeheng Wang

Academic Editor

PLOS One

**Journal Requirements:**

1. When submitting your revision, we need you to address these additional requirements.

Please ensure that your manuscript meets PLOS ONE's style requirements, including those for file naming. The PLOS ONE style templates can be found at

https://journals.plos.org/plosone/s/file?id=wjVg/PLOSOne_formatting_sample_main_body.pdf and

https://journals.plos.org/plosone/s/file?id=ba62/PLOSOne_formatting_sample_title_authors_affiliations.pdf

2. Please note that PLOS One has specific guidelines on code sharing for submissions in which author-generated code underpins the findings in the manuscript. In these cases, we expect all author-generated code to be made available without restrictions upon publication of the work. Please review our guidelines at https://journals.plos.org/plosone/s/materials-and-software-sharing#loc-sharing-code and ensure that your code is shared in a way that follows best practice and facilitates reproducibility and reuse.

3. Thank you for stating the following financial disclosure:

VZ and JS acknowledges funding from Special Focus Area Personalized Health and Related Technologies (SFA PHRT) iDoc Project 2023-381.

Please state what role the funders took in the study.  If the funders had no role, please state: "The funders had no role in study design, data collection and analysis, decision to publish, or preparation of the manuscript."

If this statement is not correct you must amend it as needed.

Please include this amended Role of Funder statement in your cover letter; we will change the online submission form on your behalf.

4. We notice that your supplementary table is included in the manuscript file. Please remove and upload it with the file type 'Supporting Information'. Please ensure that each Supporting Information file has a legend listed in the manuscript after the references list.

5. We note that Figure S1 in your submission contain copyrighted images. All PLOS content is published under the Creative Commons Attribution License (CC BY 4.0), which means that the manuscript, images, and Supporting Information files will be freely available online, and any third party is permitted to access, download, copy, distribute, and use these materials in any way, even commercially, with proper attribution. For more information, see our copyright guidelines: http://journals.plos.org/plosone/s/licenses-and-copyright.

We require you to either present written permission from the copyright holder to publish these figures specifically under the CC BY 4.0 license, or remove the figures from your submission:

a. You may seek permission from the original copyright holder of Figure S1 to publish the content specifically under the CC BY 4.0 license.

We recommend that you contact the original copyright holder with the Content Permission Form (http://journals.plos.org/plosone/s/file?id=7c09/content-permission-form.pdf) and the following text:

“I request permission for the open-access journal PLOS ONE to publish XXX under the Creative Commons Attribution License (CCAL) CC BY 4.0 (http://creativecommons.org/licenses/by/4.0/). Please be aware that this license allows unrestricted use and distribution, even commercially, by third parties. Please reply and provide explicit written permission to publish XXX under a CC BY license and complete the attached form.”

Please upload the completed Content Permission Form or other proof of granted permissions as an "Other" file with your submission.

In the figure caption of the copyrighted figure, please include the following text: “Reprinted from [ref] under a CC BY license, with permission from [name of publisher], original copyright [original copyright year].”

b. If you are unable to obtain permission from the original copyright holder to publish these figures under the CC BY 4.0 license or if the copyright holder’s requirements are incompatible with the CC BY 4.0 license, please either i) remove the figure or ii) supply a replacement figure that complies with the CC BY 4.0 license. Please check copyright information on all replacement figures and update the figure caption with source information. If applicable, please specify in the figure caption text when a figure is similar but not identical to the original image and is therefore for illustrative purposes only.

6. If the reviewer comments include a recommendation to cite specific previously published works, please review and evaluate these publications to determine whether they are relevant and should be cited. There is no requirement to cite these works unless the editor has indicated otherwise.

[Note: HTML markup is below. Please do not edit.]

Reviewers' comments:

**Comments to the Author**

1. Is the manuscript technically sound, and do the data support the conclusions?

Reviewer #1: Yes

Reviewer #2: Partly

Reviewer #3: Partly

Reviewer #4: Yes

**********

2. Has the statistical analysis been performed appropriately and rigorously?

Reviewer #1: Yes

Reviewer #2: No

Reviewer #3: N/A

Reviewer #4: Yes

**********

3. Have the authors made all data underlying the findings in their manuscript fully available?

The PLOS Data policy

Reviewer #1: Yes

Reviewer #2: Yes

Reviewer #3: Yes

Reviewer #4: Yes

**********

4. Is the manuscript presented in an intelligible fashion and written in standard English?

Reviewer #1: Yes

Reviewer #2: Yes

Reviewer #3: Yes

Reviewer #4: Yes

**********

**Reviewer #1:**  This is a meticulously conducted and highly transparent study that addresses a critical challenge in polarization-resolved second harmonic generation microscopy. The authors honestly report their experimental difficulties in achieving perfect linear polarization control, despite implementing a state-of-the-art waveplate compensation method on a commercial microscope. Their systematic documentation of hardware modifications, careful measurement of residual ellipticity (up to 0.25), and insightful simulation linking the problem to wavelength-dependent birefringence and broad femtosecond bandwidth are particularly commendable. The conclusion that conventional compensation may be fundamentally limited for broadband pulses is an important cautionary insight for the field. Moreover, their decision to share open-source analysis code and simulation files sets an excellent example of reproducible and collaborative science. This work will undoubtedly guide future researchers toward alternative strategies, such as narrow-bandwidth picosecond sources or specimen rotation, and prevents many from wasting effort on unattainable compensation.

**Reviewer #2:**  1. Summary of the Manuscript

This manuscript investigates a fundamental limitation of waveplate-based polarization compensation in polarization-resolved second harmonic generation (pSHG) microscopy. The authors implement an established automated compensation method — a rotating half-waveplate (HWP) combined with a compensating quarter-waveplate (QWP) — on a commercial Leica TCS SP8 MP multiphoton microscope, building upon the approach of Romijn et al. (2018). Despite careful hardware implementation and significantly improved acquisition speed (360 ms per measurement vs. approximately 17 s in the original implementation), the authors consistently observe residual polarization ellipticity with amplitudes up to 0.25 that persists even under optimal waveplate configurations.

Through Jones matrix simulations incorporating polychromatic illumination, the authors propose that the residual ellipticity arises from the interaction between wavelength-dependent dichroic mirror birefringence and the broad spectral bandwidth (10-20 nm FWHM) of femtosecond laser pulses. Their polychromatic simulations qualitatively reproduce the observed periodic ellipticity variation (simulated amplitude ~0.13 vs. experimental ~0.25). The authors conclude with practical recommendations including specimen rotation or narrowband picosecond laser sources as alternatives for precision polarization control. Open-source analysis code, simulation files, and experimental datasets are provided.

2. Major Comments

The following major issues require substantive revision and should be addressed before the manuscript can be considered for acceptance.

Major Comment 1: The Central Mechanistic Claim Lacks Direct Experimental Validation

The paper's core hypothesis — that residual ellipticity arises from wavelength-dependent dichroic mirror birefringence combined with femtosecond pulse bandwidth — is supported only by simulation, not by direct measurement. The authors acknowledge that direct measurement of the dichroic mirror retardance spectrum 'would be required for quantitative validation but is beyond the scope of this work.' This is a significant limitation for a paper whose title asserts this mechanism as the explanation. The manuscript never actually measures the spectral retardance of the specific dichroic mirror in the Leica SP8 system; instead it borrows values from Schon et al. (2008), which were obtained on a different system. Without at least a qualitative experimental test — for example, comparing ellipticity residuals when using a narrowband (picosecond) versus broadband (femtosecond) source at the same center wavelength — the causal claim remains speculative. The authors should either perform this comparison, characterize their dichroic mirror directly using ellipsometry or the Mueller matrix method, or substantially moderate the language of their title and conclusions to reflect that the mechanism is proposed rather than demonstrated.

Major Comment 2: Quantitative Mismatch Between Simulation and Experiment Is Insufficiently Addressed

The polychromatic simulation produces residual ellipticity of approximately 0.13, while the experimental observations reach up to 0.25 — a factor of nearly two. While the authors list several possible contributing factors (simplified linear retardance model, uncertainty in the dichroic spectrum, other wavelength-dependent elements), no quantitative sensitivity analysis is performed to determine which factor is dominant or whether the proposed mechanism is even sufficient to account for the observed magnitude. This discrepancy is non-trivial: it opens the possibility that other unaccounted mechanisms — e.g., objective lens polarization aberrations, scan mirror retardance, polarization-dependent transmission in optical coatings — may contribute comparably or even dominantly. A parametric analysis showing how the simulated ellipticity amplitude changes with dichroic retardance slope, bandwidth, or the addition of further optical elements would substantially strengthen the paper's argument.

Major Comment 3: The Simulation Model Makes Unverified Simplifying Assumptions

The polychromatic Jones matrix simulation models the dichroic mirror retardance as varying linearly with wavelength from -12.9 deg to +37.1 deg over 40 nm. This linearity assumption is never justified. The reference measurement from Schon et al. (their Figure 6b) shows that dichroic retardance can exhibit strongly nonlinear, even oscillatory, spectral dependence depending on coating design. The actual spectral retardance of the Leica SP8 dichroic mirror is unknown. Furthermore, the simulation uses nine discrete wavelengths with a Gaussian weighting to represent the femtosecond pulse spectrum, but no justification is given for the adequacy of this sampling. The authors should: (1) test sensitivity of the simulation output to the retardance profile shape (linear vs. nonlinear), (2) verify convergence of the ellipticity estimate with respect to number of spectral samples, and (3) explicitly acknowledge that the borrowed dichroic values may not represent their instrument.

Major Comment 4: The Role of Objective Lens Polarization Distortions Is Dismissed Too Quickly

The authors do not specify the objective NA used, nor do they provide any quantification of its contribution to the observed ellipticity. Given that the paper attributes residual ellipticity specifically to the dichroic-bandwidth interaction, the absence of a systematic assessment of the objective lens contribution — particularly since measurements at the entrance port (ellipticity < 0.05) vs. sample plane (up to 0.25) show a significant increase through the microscope body — weakens the attribution. The authors should at minimum report the objective NA, reference appropriate literature on objective-induced polarization effects, and discuss whether this contribution has been ruled out or quantified.

Major Comment 5: Statistical Characterization of Ellipticity Measurements Is Absent

The paper presents ellipticity curves and maps as single-instance measurements without any statistical characterization — no error bars, no repeated measurement variability, no confidence intervals. The authors mention that their polarization analyzer takes 360 ms per measurement and that validation was done against a power meter (S4 Fig), but the reproducibility of the two-dimensional ellipticity maps (requiring 30 minutes of acquisition) is never assessed. Questions that remain unanswered include: How stable is the laser polarization state over 30 minutes? Does the ellipticity map change between acquisition runs? What is the measurement noise floor for the 2D maps? The authors should provide repeated measurements of at least the compensation curve (Fig. 3B) with appropriate error characterization, and discuss measurement stability over the full acquisition duration.

Major Comment 6: The Practical Significance Threshold Is Not Adequately Justified

The authors state that 'for some quantitative polarization-resolved measurements, a residual ellipticity of 0.25 may be acceptable depending on the required measurement precision.' This statement risks being misleading without context. No quantitative analysis is provided linking a given ellipticity value to resulting errors in downstream SHG analysis parameters (e.g., orientation angle error, molecular order parameter bias). The paper would be considerably strengthened by including either an analytical or numerical estimate of how ellipticity of 0.06-0.25 propagates into errors in the polarization-dependent SHG intensity ratio or fitted structural parameters. A worked example or sensitivity analysis connecting ellipticity magnitude to SHG measurement error is strongly recommended.

Major Comment 7: Alternative Explanations for the Two-Peak Residual Pattern Are Not Ruled Out

The two-peak periodic structure of the residual ellipticity along the compensation curve (Fig. 3B) is attributed to the polychromatic mechanism. However, this same two-peak structure could in principle arise from: (a) imperfect waveplate retardance calibration (the HWP is specified at 0.516 lambda instead of 0.5 lambda), (b) waveplate angular misalignment or wobble during rotation, (c) beam walk-off during HWP rotation, or (d) residual effects from the five silver mirrors not captured by the assumed model. The monochromatic simulation already shows that non-ideal waveplate retardances introduce asymmetry (Fig. 2B). The authors should explicitly demonstrate — through simulation or experimental controls — that these alternative explanations are insufficient to account for the observed residual ellipticity pattern before attributing it to the spectral bandwidth mechanism.

Major Comment 8: The Proposed Alternative Solutions Are Insufficiently Analyzed

The conclusions recommend specimen rotation and picosecond laser sources as alternatives, but neither is analyzed quantitatively in terms of its own practical limitations. Picosecond lasers reduce SHG efficiency (signal scales as peak power squared for pulsed excitation), which could be significant for weakly scattering biological specimens. The authors should provide at least a brief quantitative estimate of the SHG signal penalty expected when switching from femtosecond to picosecond pulses at the same average power, and discuss whether this tradeoff is acceptable for their targeted applications. Similarly, specimen rotation introduces translation-rotation coupling that the authors acknowledge but do not quantify in terms of required mechanical precision.

3. Minor Comments

The following minor issues should be addressed during revision:

• Title precision: The phrase 'may arise' is appropriately cautious, but given that the paper focuses on a single proposed mechanism without ruling out alternatives, consider further qualifying the claim (e.g., specifying 'in part' or 'under certain conditions').

• Waveplate ordering clarification (p. 2): The practical implications of the QWP-HWP vs. HWP-QWP ordering difference for the compensation mathematics should be stated explicitly in the main text rather than relegated to the Acknowledgments, as it is methodologically relevant.

• Figure 2B: The caption states the light blue curve shows ellipticity reduced to zero, but the minimum and maximum ellipticity achieved with this configuration should be stated explicitly as numerical values.

• Figure 3B: The legend includes 'HWP+QWP*' but the caption does not explain what the asterisk denotes. This should be clarified.

• Figure 4D: The monochromatic curve shows small oscillations near zero that appear to be numerical fitting artifacts. Their origin should be acknowledged and explained.

• Missing objective specification: The numerical aperture and model of the objective lens used in all measurements must be stated in the Materials and Methods section. This is a critical experimental parameter for interpreting polarization results.

• Laser bandwidth discrepancy: A simulation at the actual specified bandwidth (12.5 nm FWHM) should be included alongside the 20 nm case, as it would provide a more direct comparison to the experimental system.

• Detection sensitivity at low ellipticity (p. 4): Since the entrance port measurements show ellipticity values approaching the photodiode's ~0.074 reliability threshold, this limitation should be discussed more prominently in relation to the entrance port characterization.

• S1 Table parameter discrepancy: The fitted HWP angle theta_0 varies by 7.5 deg between measurement locations. This discrepancy should be discussed more carefully, as it may indicate that the Romijn et al. model is more substantially misspecified than the text implies.

• AI assistance disclosure: The authors note use of Claude 4.5 Sonnet for manuscript preparation. PLOS ONE's editorial policies on AI use should be verified to ensure full compliance with the journal's current requirements.

• Equation self-containedness: Equations 1-3 from Romijn et al. are used without re-deriving or restating them. At minimum, the physical meaning of each parameter in those equations should be briefly defined in the text for the paper to be self-contained.

• References: Reference 6 (Cisek et al., 2023) is a book chapter that may be difficult to access. Where possible, peer-reviewed journal articles should be prioritized for core methodological citations.

**Reviewer #3:**  Review Comments to the Author

The manuscript presents a careful experimental and computational investigation of residual ellipticity in waveplate-compensated polarization-resolved SHG microscopy. The study is technically sound, well-structured, and demonstrates a strong commitment to reproducibility through the provision of open-source code, datasets, and hardware designs. The work is relevant to the nonlinear microscopy community and addresses a practically important issue in polarization control.

However, several aspects require clarification and strengthening before the manuscript can be considered for publication.

First, the novelty of the work should be more clearly articulated. While the study is well executed, polarization distortions and wavelength-dependent birefringence in optical systems are already known. The authors should explicitly define what new insight is provided beyond prior work (e.g., Romijn et al.), particularly whether the main contribution lies in identifying the magnitude of residual ellipticity, demonstrating the limitations of compensation, or linking the effect to femtosecond laser bandwidth.

Second, the central conclusion—that residual ellipticity arises from the combination of spectral bandwidth and wavelength-dependent dichroic retardance—is supported primarily by simulations and qualitative agreement with experiments. There is no direct experimental validation of this hypothesis. The authors are encouraged to either (i) provide additional experimental evidence (e.g., bandwidth filtering or comparison with a narrower linewidth source), or (ii) moderate the strength of their claims and clearly frame this as a plausible explanation rather than a definitive conclusion.

Third, the simulation model appears simplified (e.g., linear retardance assumption, limited spectral sampling), and does not fully reproduce the experimentally observed amplitude of ellipticity. A more critical discussion of these limitations, along with sensitivity analysis or justification of modeling assumptions, would strengthen the manuscript.

Fourth, the study is conducted on a single microscope system. The authors should discuss the generalizability of their findings to other systems and optical configurations, and clarify whether the observed limitations are expected to be universal or system-dependent.

Fifth, the practical impact of the reported ellipticity values (up to 0.25) is not quantitatively analyzed. Since polarization-resolved SHG is often used for structural inference, it would be valuable to include a clearer discussion—or preferably a quantitative example—of how such residual ellipticity affects downstream measurements or interpretation.

Finally, while the manuscript is generally well written, some sections (particularly in Materials and Methods) are overly detailed and could be streamlined or moved to supplementary materials to improve readability.

In summary, the manuscript is promising and technically solid, but requires clarification of novelty, stronger support (or more cautious interpretation) of the main claim, and improved discussion of limitations and impact. With these revisions, the work would make a useful contribution to the field.

**Reviewer #4:**  This manuscript investigates the issue of residual ellipticity in waveplate-compensated polarization-resolved second harmonic generation microscopy, proposing that the spectral bandwidth of the femtosecond laser is the primary limiting factor. Upon review, the manuscript exhibits five major flaws concerning experimental validation, theoretical simulation, and model rigor.

First, the core hypothesis lacks direct experimental data support. The authors claim that residual ellipticity originates from the combination of the wavelength-dependent retardance of the dichroic mirror and the broadband spectrum of the laser. However, they explicitly admit to not measuring the actual retardance spectrum of the Leica microscope dichroic mirror used, instead directly inferring it from referenced data. This unverified substitution weakens the direct persuasiveness of the conclusions.

Second, there is a significant quantitative discrepancy between the Jones matrix simulation results and the experimental data. The residual ellipticity amplitude derived from the polychromatic light simulation is only around 0.13, whereas the actual experimental measurement is as high as 0.25. Although the authors mention this difference, they fail to further refine the physical model to bridge this gap, indicating that the current theoretical explanation is quantitatively incomplete.

Third, the parameter settings for the theoretical simulations are disconnected from the actual experimental equipment. The Mai Tai femtosecond laser used in the experiment has a nominal bandwidth of 12.5 nm, yet the authors employed a bandwidth of 20 nm in the simulation. The practice of artificially relaxing parameters to highlight spectral effects lacks rigor. Furthermore, even with the expanded bandwidth parameter, the simulated polarization distortion remains far below the true experimental value.

Fourth, the established optical system model is overly simplified and ignores key optical components. The simulation only includes waveplates, five metal mirrors, and a dichroic mirror, completely omitting the high numerical aperture objective lens. High numerical aperture objectives are known to cause severe polarization distortions. Excluding the most critical imaging component of the microscope results in a simulation environment that fails to reflect the complexity of the optical path at the sample plane.

Fifth, there are issues with the applicability of the fitting model, resulting in fitting parameters that lack a reasonable physical basis. According to the supplementary data, the initial angle parameter of the half-wave plate exhibits a massive shift from 18.07 degrees to 25.61 degrees between the entrance port and the sample plane. Since the physical mounting position of the half-wave plate remained unchanged, this parameter jump is illogical. The authors simply attribute this to the model's inability to fully capture the sources of residual ellipticity, exposing a lack of robustness in their mathematical fitting model when handling real system errors.

**********

what does this mean?). If published, this will include your full peer review and any attached files.

If you choose “no”, your identity will remain anonymous but your review may still be made public.

**Do you want your identity to be public for this peer review?**  For information about this choice, including consent withdrawal, please see our Privacy Policy

Reviewer #1: **Yes:**  Chen yu Zhao

Reviewer #2: **Yes:**  Muhammad Zunnurain

Reviewer #3: No

Reviewer #4: No

**********

[NOTE: If reviewer comments were submitted as an attachment file, they will be attached to this email and accessible via the submission site. Please log into your account, locate the manuscript record, and check for the action link "View Attachments". If this link does not appear, there are no attachment files.]

To ensure your figures meet our technical requirements, please review our figure guidelines: https://journals.plos.org/plosone/s/figures

You may also use PLOS’s free figure tool, NAAS, to help you prepare publication quality figures: https://journals.plos.org/plosone/s/figures#loc-tools-for-figure-preparation.

NAAS will assess whether your figures meet our technical requirements by comparing each figure against our figure specifications.

Attachment Submitted filename: Major & Minor commetns.docx

Attachment Submitted filename: renamed_a26b7.pdf

# Author comment: Author response to Decision Letter 1

**DOI:** 10.1371/journal.pone.0357256.r002

14 Aug 2026

See Response to Reviewers.pdf attached.

Attachment Submitted filename: Response to Reviewers.pdf

# Peer review: Decision Letter 1

**DOI:** 10.1371/journal.pone.0357256.r003

**Authors:** Zeheng Wang

27 Oct 2026

Persistent residual ellipticity in waveplate-compensated polarization-resolved SHG microscopy on a commercial multiphoton microscope

PONE-D-26-06443R1

Dear Dr. Nguyen,

We’re pleased to inform you that your manuscript has been judged scientifically suitable for publication and will be formally accepted for publication once it meets all outstanding technical requirements.

Within one week, you’ll receive an e-mail detailing the required amendments. When these have been addressed, you’ll receive a formal acceptance letter and your manuscript will be scheduled for publication.

An invoice will be generated when your article is formally accepted. Please note, if your institution has a publishing partnership with PLOS and your article meets the relevant criteria, all or part of your publication costs will be covered. Please make sure your user information is up-to-date by logging into Editorial Manager at Editorial Manager® and clicking the ‘Update My Information' link at the top of the page. For questions related to billing, please contact billing support.

If your institution or institutions have a press office, please notify them about your upcoming paper to help maximize its impact. If they’ll be preparing press materials, please inform our press team as soon as possible -- no later than 48 hours after receiving the formal acceptance. Your manuscript will remain under strict press embargo until 2 pm Eastern Time on the date of publication. For more information, please contact onepress@plos.org.

Kind regards,

Zeheng Wang

Academic Editor

PLOS One

Additional Editor Comments (optional):

Reviewers' comments:

Reviewer's Responses to Questions

**Comments to the Author**

Reviewer #4: All comments have been addressed

**********

2. Is the manuscript technically sound, and do the data support the conclusions?

Reviewer #4: Yes

**********

3. Has the statistical analysis been performed appropriately and rigorously?

Reviewer #4: Yes

**********

4. Have the authors made all data underlying the findings in their manuscript fully available?

The PLOS Data policy

Reviewer #4: Yes

**********

5. Is the manuscript presented in an intelligible fashion and written in standard English?

Reviewer #4: Yes

**********

Reviewer #4: I have carefully reviewed your revised manuscript, the track-changes version, and your point-by-point responses to the four reviewers. I am pleased to inform you that your manuscript is now accepted for publication in PLOS ONE.

Congratulations on this meticulous and open piece of work. Your findings will be of significant value to researchers working in nonlinear polarization optics and biomedical microscopy.

**********

what does this mean?). If published, this will include your full peer review and any attached files.

If you choose “no”, your identity will remain anonymous but your review may still be made public.

**Do you want your identity to be public for this peer review?**  For information about this choice, including consent withdrawal, please see our Privacy Policy

Reviewer #4: **Yes:**  DENG ZEHUI

**********

Attachment Submitted filename: renamed_aae80.pdf

# Editor report: Acceptance letter

**DOI:** 10.1371/journal.pone.0357256.r004

**Authors:** Zeheng Wang

PONE-D-26-06443R1

PLOS One

Dear Dr. Nguyen,

I'm pleased to inform you that your manuscript has been deemed suitable for publication in PLOS One. Congratulations! Your manuscript is now being handed over to our production team.

At this stage, our production department will prepare your paper for publication. This includes ensuring the following:

* All references, tables, and figures are properly cited

* All relevant supporting information is included in the manuscript submission,

* There are no issues that prevent the paper from being properly typeset

You will receive further instructions from the production team, including instructions on how to review your proof when it is ready. Please keep in mind that we are working through a large volume of accepted articles, so please give us a few days to review your paper and let you know the next and final steps.

Lastly, if your institution or institutions have a press office, please let them know about your upcoming paper now to help maximize its impact. If they'll be preparing press materials, please inform our press team within the next 48 hours. Your manuscript will remain under strict press embargo until 2 pm Eastern Time on the date of publication. For more information, please contact onepress@plos.org.

You will receive an invoice from PLOS for your publication fee after your manuscript has reached the completed accept phase. If you receive an email requesting payment before acceptance or for any other service, this may be a phishing scheme. Learn how to identify phishing emails and protect your accounts at https://explore.plos.org/phishing.

If we can help with anything else, please email us at customercare@plos.org.

Thank you for submitting your work to PLOS One and supporting open access.

Kind regards,

PLOS One Editorial Office Staff

on behalf of

Dr. Zeheng Wang

Academic Editor

PLOS One
